# Volatilomic Profiling of Early-Stage Biotransformation Enables Identification of Diagnostic Volatiles in *Escherichia coli*-Contaminated Chicken Meat

**DOI:** 10.3390/foods15183198

**Published:** 2026-09-10

**Authors:** Jing Jin, Yajing Guo, Leijie Wang, Qingqing Liu, Wei Cao, Yin Wang

**Affiliations:** College of Food Science and Technology, Northwest University, 229 North TaiBai Road, Xi’an 710069, China; 202323976@stumail.nwu.edu.cn (J.J.); 202434213@stumail.nwu.edu.cn (Y.G.); 202434221@stumail.nwu.edu.cn (L.W.); liuqq@nwu.edu.cn (Q.L.); caowei@nwu.edu.cn (W.C.)

**Keywords:** VOCs, *Escherichia coli*, HS-GC-IMS, HS-SPME-GC-MS

## Abstract

*E. coli* inoculation in food can be accompanied by a series of biochemical changes, during which both microbial activity and food matrix degradation may contribute to the formation of volatile organic compounds (VOCs). The composition and variation in these VOCs depend on the food, the contaminating microorganisms, and storage conditions. This study investigated VOC evolution in *E. coli*-inoculated chicken meat over 48 h using HS-GC-IMS and HS-SPME-GC-MS, detecting 57 and 50 VOCs, respectively. HS-GC-IMS showed advantages in detecting low-molecular-weight compounds. Through OPLS-DA and KEGG-based pathway analysis, several stage-associated VOCs were identified as candidate VOCs, including benzaldehyde (early stage of the 48 h incubation period), 3-methylbutanol and 3-(methylthio) propanal (middle stage of the 48 h incubation period), and 3-methyl-3-buten-1-ol, phenethyl alcohol, and methyl N-hydroxybenzenecarboximidate (late stage of the 48 h incubation period). Further analysis suggested that the formation of these VOCs may involve lipid oxidation, amino acid degradation, and microbial metabolism, providing a basis for the further validation of VOC-based approaches for monitoring changes in *E. coli*-inoculated poultry meat.

## 1. Introduction

Foodborne pathogens posed a significant threat to global public health and safety, resulted in approximately 600 million illness cases, 420,000 fatalities [1], and 33 million disability-adjusted life years lost annually worldwide. China’s consumption of chicken meat is expected to increase to 37.8% by 2025 as a result of its high protein and low-fat content [2]. Despite its high nutritional value, chicken meat is highly susceptible to microbial contamination during processing, storage, and transportation. Moreover, its quality continues to deteriorate during storage as a result of both microbial metabolism and non-microbial reactions, such as lipid oxidation, proteolysis, and other matrix-related changes. Recent studies have further demonstrated that VOC profiles undergo dynamic changes during chicken meat deterioration, with specific aldehydes, ketones, and alcohols showing potential as indicators of quality degradation [3]. Collectively, these processes can markedly alter the volatile profile of chicken meat.

Conventional culture-based methods remain reliable for foodborne pathogen detection but are limited by lengthy, multistep procedures. Microbial metabolism can alter food volatile organic compounds (VOCs) profiles, producing alcohols, aldehydes, ketones, acids, and sulfur-containing compounds [4]. However, as many VOCs may also originate from endogenous reactions or general spoilage, VOC patterns are better suited for rapid screening and monitoring microbial-associated changes than as direct species-specific biomarkers.

In recent years, there has been a growing array of food analysis methods utilizing VOCs. Commonly applied techniques include electronic nose technology (E-nose), headspace solid-phase microextraction-gas chromatography–mass spectrometry (HS-SPME-GC-MS), gas chromatography–olfactometry–mass spectrometry (GC-O-MS) [5], and headspace gas chromatography–ion mobility spectrometry (HS-GC-IMS) [6]. The E-nose enables rapid evaluation of overall VOC fingerprints; however, it provides limited information for the accurate identification of individual volatile compounds [7,8]. HS-SPME-GC-MS exhibits excellent separation capability and structural identification accuracy for VOCs in complex food matrices, although its sensitivity toward certain low-molecular-weight compounds remains relatively limited [9,10]. In contrast, HS-GC-IMS enables rapid VOC fingerprint visualization and sensitive detection of many volatile compounds, whereas compound annotation remains dependent on the availability and completeness of reference databases [11]. Thus, the two analytical platforms provide complementary rather than redundant information and may jointly offer a more comprehensive characterization of dynamic VOC changes in complex food matrices (Table 1).

Previous studies have identified a wide range of VOCs associated with *E. coli* under culture-based, clinical, diagnostic, and other experimental conditions [14,15,16]. These studies have demonstrated that VOC analysis provides valuable information for bacterial characterization. However, VOC profiles are highly dependent on substrate composition, growth conditions, incubation time, background microbiota, and analytical methods, limiting their direct extrapolation to complex food matrices such as chicken meat. Moreover, the temporal evolution of *E. coli*-associated VOCs in chicken and the complementary capabilities of HS-GC-IMS and HS-SPME-GC-MS remain insufficiently understood. Addressing these gaps is essential for distinguishing matrix-related volatile changes from reproducible VOC patterns associated with bacterial inoculation.

HS-SPME-GC-MS and HS-GC-IMS were integrated to characterize the temporal evolution of VOCs and identify candidate VOCs associated with *E. coli* inoculation in chicken meat over 48 h. KEGG-based pathway mapping was further used to explore putative metabolic routes associated with selected VOCs. The complementary information provided by the two analytical platforms may facilitate a more comprehensive understanding of VOC changes and provide a basis for the future development of VOC-based approaches for monitoring microbial contamination in poultry products.

## 2. Materials and Methods

### 2.1. Bacterial Strains and Chemicals

*E. coli* ATCC 25922 was stored at −80 °C in our lab. LB agar and LB broth were supplied by Beijing Auboxing Co., Ltd. (Beijing, China). Fresh slaughtered chicken was acquired from supermarket (Xi’an, China). 2-Octanol (98%) was acquired from Sigma-Aldrich Company, Ltd. (Shanghai, China). The C_7_-C_40_ n-alkane mixture was acquired from Yuanye Bio-Technology Co., Ltd. (Shanghai, China). Pure water was produced by the MilliQ purification system (Millipore, Waltham, MA, USA).

### 2.2. Preparation of Chicken Samples

Two freshly slaughtered chilled chickens of the same breed and commercial batch were purchased from a Renrenle supermarket in Xi’an, Shaanxi Province, China, in March 2026. The chickens were slaughtered on the day of purchase, wrapped in plastic film, and transported to the laboratory under chilled conditions (approximately 4 °C). Under aseptic conditions, the skin was removed, and breast meat was collected from the same anatomical region of each chicken. Spatially separated portions (5.0 ± 0.8 g) were excised from the breast muscle, with a small distance maintained between adjacent sampling sites to minimize overlap between portions. One chicken was assigned to the higher-inoculation group (M), whereas the other was assigned to the lower-inoculation group (L). For each inoculation group, individual breast portions were assigned to the six incubation times (0, 4, 8, 12, 24, and 48 h). An additional uninoculated breast portion processed under the same experimental conditions was used as the negative control (CK). Each 5.0 ± 0.8 g portion was rinsed five times with sterile water to reduce surface-associated background microorganisms and was then aseptically divided into three approximately equal parallel subsamples. These three subsamples originated from the same parent breast portion and were therefore treated as parallel technical subsamples rather than independent biological replicates. The subsamples were subsequently subjected to the same inoculation and analytical procedures within the corresponding experimental group. Single colonies of *E. coli* ATCC 25922 were suspended in 1 mL of sterile saline (0.85%, *w*/*v*). The viable concentration of the original bacterial suspension was approximately 3.56 × 10^7^ CFU/mL, as determined by plate counting before inoculation. The bacterial suspension was subsequently serially diluted to 10^−4^ and 10^−6^, which were designated as Group M and Group L, respectively. Two hundred microliters of the suspension were separately inoculated into chicken meat. To simulate ambient-temperature handling and temporary storage conditions relevant to fresh chicken meat sold in traditional retail markets, rather than conventional refrigerated storage, samples were stored at 20 °C for 0, 4, 8, 12, 24, and 48 h. This condition was used as a controlled ambient-temperature spoilage scenario to characterize the temporal changes in VOC profiles. At each sampling time, 3 mL of sterile saline (0.85%, *w*/*v*) was added to each tube, and the chicken sample was thoroughly rinsed and shaken to obtain the rinsate. For VOC analysis, 3 mL of sterile saline rinsate obtained from each chicken sample was collected. The rinsates collected at 0, 4, and 8 h were temporarily stored at −20 °C until VOC analysis was initiated at the 12 h sampling point, corresponding to approximate frozen-storage durations of 12, 8, and 4 h, respectively. Each frozen rinsate underwent only one freeze–thaw cycle before analysis. Samples collected from 12 h onward were analyzed without this pre-analysis frozen-storage step. For each inoculation condition, three parallel subsamples derived from the same 5 g treatment portion were independently inoculated and processed. These subsamples were regarded as parallel within-sample replicates rather than independent biological replicates. The individual chicken represented the independent biological source at each sampling time, while the M and L portions constituted paired treatment samples derived from the same chicken.

### 2.3. HS-GC-IMS Analysis

Volatile compounds from *E. coli*-inoculated chicken samples were analyzed using HS-GC-IMS (FlavourSpec^®^, Gesellschaft für Analytische Sensorsysteme mbH [G.A.S.], Dortmund, Germany). A 500 μL aliquot of the resulting rinsate was collected for subsequent HS-GC-IMS analysis. It was incubated at 40 °C for 15 min in headspace vials. Separation employed an FS-SE-54-CB-1 capillary column (15 m × 0.53 mm i.d., 1 μm film thickness; CS-Chromatographie Service GmbH, Langerwehe, Germany) at 40 °C with high-purity N2 (99.999%) as drift gas at 150 mL/min at 45 °C. The GC flow rate was initially 2.0 mL/min (2 min), then linearly increased to 100 mL/min over 20 min [13]. All analyses were performed in triplicate.

HS-GC-IMS data were analyzed utilizing Laboratory Analytical Viewer software (LAV, G.A.S., Dortmund, Germany). C_4_-C_9_ n-ketone standard was chosen as the carbon standard and analyzed by GC-IMS following the sample determination procedure. LRI index of the target compound was determined based on the retention time of the isolated compound’s peak The qualitative investigation of the chemical was conducted by comparing the RI index from the NIST library and utilizing IMS database retrieval software acquired from G.A.S. (Dortmund, Germany). GC-IMS data were processed using Laboratory Analytical Viewer (LAV; G.A.S., Dortmund, Germany). The Reporter module was used to inspect the two- and three-dimensional spectra. Retention indices (RI) were calculated using external standards, and VOCs were identified by matching RI and IMS drift time with the GC-IMS library. The Gallery Plot module was used to generate VOC fingerprints from reproducibly detected signal peaks. Peaks were retained when they were clearly distinguishable from the local background and occurred at consistent retention-time and relative drift-time positions across replicate measurements; sporadic or poorly resolved signals were excluded. No additional statistical pre-selection based on statistical significance, VIP values, or fold changes was performed before Gallery Plot generation.

### 2.4. HS-SPME-GC-MS Analysis

Each sample was mixed with 12 mL of sterile distilled water and vortexed for 30 s. Five milliliters of the resulting diluted sample were placed into a 20 mL amber glass screw-capped headspace vial, and 9 μL of 2-octanol (0.1638 μg/μL, corresponding to a final concentration of 294.84 µg/L in the vial) was added as an internal standard.

Samples were incubated in a shaker bath at 60 °C for 20 min, then extracted using a 50/30 µm DVB/CAR/PDMS SPME fiber (Agilent Technologies, Santa Clara, CA, USA) for 30 min at 45 °C. Helium was used as a carrier gas at a constant flow rate of 1.6 mL/min (R). Extraction conditions were as follows: equilibrium at 60 °C for 20 min; sample penetration at 35 mm depth and 20 mm/s velocity; extraction duration of 30 min at 45 °C; and stirring at 250 rpm. The temperature ramping protocol was: 40 °C for 3 min, increased to 70 °C at 3 °C/min, then to 180 °C at 5 °C/min, and finally to 230 °C at 10 °C/min with a 5 min hold. Detection parameters were: injection port temperature 250 °C, electronic ion source 230 °C, quadrupole detector 150 °C, and scanning range of 30–550. All samples were performed in triplicate.

HS-SPME-GC-MS data were processed using MassHunter Qualitative Analysis software 10.0 (Agilent Technologies, Santa Clara, CA, USA). VOCs were identified by comparing the acquired mass spectra with those in the NIST17 mass spectral library and by considering the corresponding retention indices. C_7_-C_40_ n-alkane standards were chosen as the carbon standards and analyzed following the sample determination protocol. LRI values of volatile compounds were calculated based on the retention times of the separated peaks using the specified formula [17]. Tentative identification was based on agreement between the mass spectral library match and retention-index information, with |RI-LRI| < 20 used as the RI acceptance criterion [18]. Because authentic standards were not used to confirm all detected compounds, the reported compound identities should be considered tentative unless otherwise specified.

$$LRI=100\times \left[{(\log}_{10}^{x_{i}}-\log_{10}^{x_{n}})/{(\log}_{10}^{x_{n+1}}-\log_{10}^{x_{n}})+n\right]$$

*x_i_* indicates the retention time of the target compound, whereas *x_n_* and *x_n+_*_1_ represent the retention durations of the adjacent alkane standards preceding and succeeding the target compound.

Semi-quantitative estimation of VOCs was performed using 2-octanol as a single internal standard. Because compound-specific calibration curves, response factors, recovery tests, and LOD/LOQ determinations were not established, the calculated values were expressed as 2-octanol equivalents and should be interpreted as semi-quantitative estimates rather than absolute concentrations. The semi-quantitative values were calculated using the following equation:

$$A_{0}=C_{0}\times A_{i}/C_{i}$$

*A*_0_ represents the substance concentration (μg/µL), while *A_i_* signifies the average peak area of the analyte. *C*_0_ indicates the concentration of the internal standard (0.1638 μg/µL), and *C_i_* denotes the average peak area of the internal standard.

### 2.5. Statistical Analysis

All results were presented as mean ± standard deviation (SD) of three parallel subsamples from the same treatment portion, with the SD reflecting within-sample experimental variability. Graphs were created using Origin Pro 2024. (OriginLab, Northampton, MA, USA). One-way ANOVA was used only as a supplementary exploratory analysis of temporal VOC variation and was not interpreted as population-level inference or used for candidate VOC selection. Correlation coefficients were calculated using SPSS Statistics 25.0 (SPSS Inc., Chicago, IL, USA). Principal Component Analysis (PCA) and Orthogonal Partial Least Squares-Discriminant Analysis (OPLS-DA) were conducted, giving significant variable (VIP) values via SIMCA 14.1 (Umetrics AB, Umeå, Sweden) software. VOC abundance data were normalized using the internal standard and Pareto-scaled before analysis. OPLS-DA models were evaluated by seven-fold cross-validation and 200 permutation tests as internal model diagnostics. VIP values were used to assist in identifying VOCs contributing to group separation. Clustering and correlation analysis of VOCs in HS-SPME-GC-MS were conducted utilizing TBtools 2.121. Putative metabolic pathways associated with selected VOCs were mapped using the Kyoto Encyclopedia of Genes and Genomes (KEGG) database (https://www.kegg.jp/; accessed on 8 July 2026).

## 3. Results and Discussion

### 3.1. Identification of Characteristic Volatiles Using HS-GC-IMS

**Figure 1:**
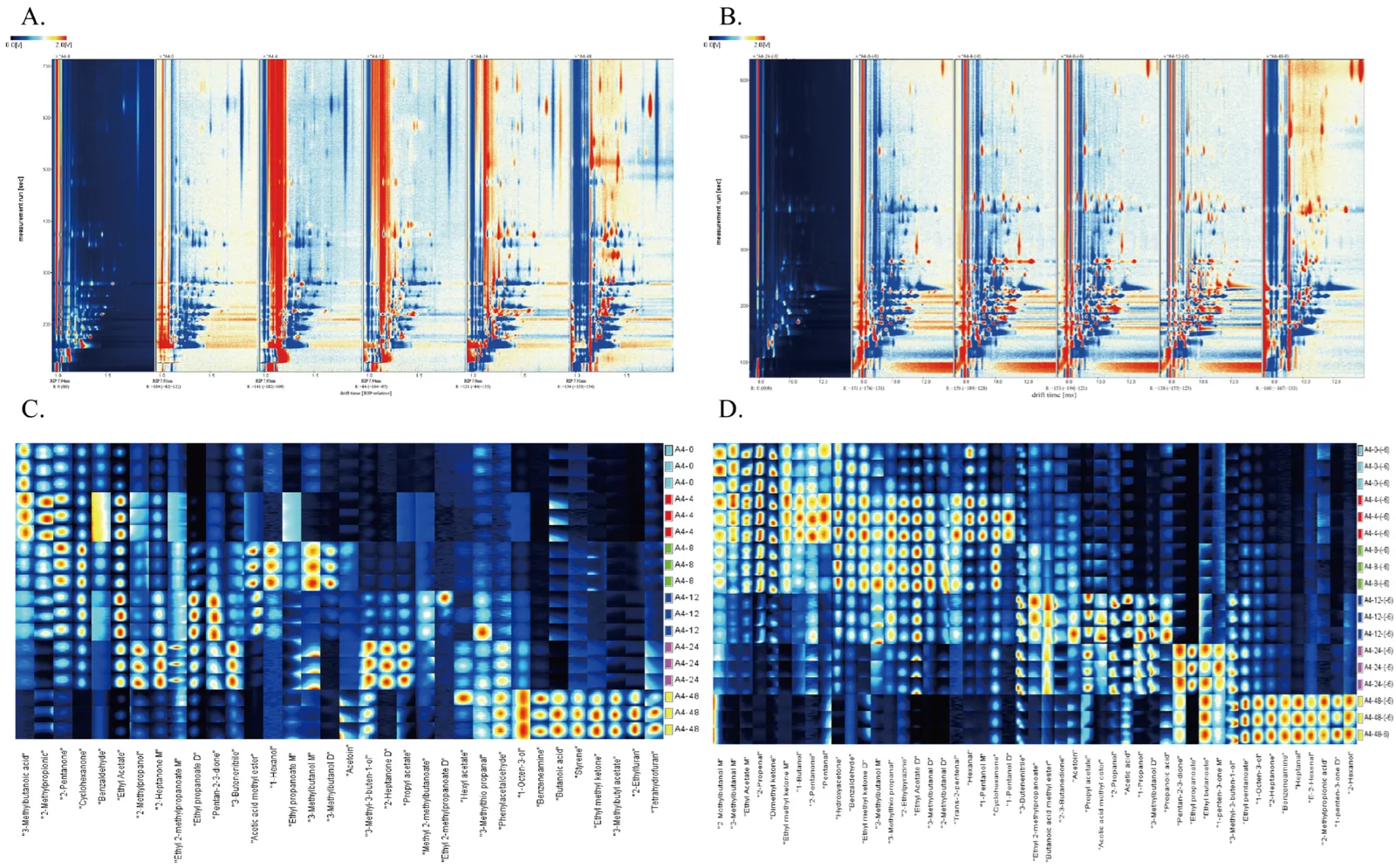
Gas chromatography–ion mobility spectrometry (GC-IMS) spectrogram of volatile compounds. (**A**) Topographic plots of *E. coli*-inoculated chicken samples during six incubation times (from left to right: 8 h, 0 h, 4 h,12 h, 24 h and 48 h); (**B**) Topographic plots of *E. coli*-inoculated chicken samples during six incubation times (from left to right: 24 h, 0 h, 4 h, 8 h, 12 h and 48 h); (**C**) Gallery plots of volatile compounds in the higher-load group during six incubation times. (**D**) Gallery plots of volatile compounds in the lower-load group during six incubation times. Panels A and B were displayed using the same color scale (0.0 M–2.0 M), with blue indicating lower signal intensity and red indicating higher signal intensity.

HS-GC-IMS was performed to comprehensively investigate the VOCs in chicken samples infected with *E. coli* at various time intervals, aiming to determine the volatile constituents and their evolving patterns. The raw data were normalized by creating a relative coordinate system for ion migration time and reactive ion peak (RIP), which created a visual topographic map of the differences based on the two-dimensional characteristics of retention time (indicating compound volatility) and ion migration time (indicating compound migration rate) (Figure 1A,B). The amplitude of the concentration change was reflected by the color depth [19]. The retention times of the majority of red signals in groups M and L varied between 100 and 400 s, whereas the drift times ranged from 1.0 to 1.5 ms and 8 to 11 ms, respectively, indicating intergroup metabolite discrepancies.

To further explore the alterations of volatile compounds in the inoculated chicken samples, the various volatile components were characterized utilizing the integrated NIST database of HS-GC-IMS. Eight compounds (3-methylbutanol, 1-pentanol, 2-methylbutanal, 3-methylbutanal, 2-heptanone, ethyl methyl ketone, 1-penten-3-one, and ethyl butanoate) were identified as possessing monomers and dimers. A total of 57 volatile compounds were identified in the spectra and categorized into 9 groups: 12 alcohols (M: 6, L: 10), 12 aldehydes (M: 5, L: 11), 12 ketones (M: 8, L: 12), 9 esters (M: 8, L: 7), 5 acids (M: 3, L: 3), 2 furans (M: 2, L: 0), 3 nitrogen-containing compounds (M: 2, L: 3), and 2 olefin species (M: 1, L: 1) (Table S1). Alcohols, aldehydes, ketones, and esters were the predominant VOCs in both sample sets. The disparity was primarily influenced by the synergistic interaction of bacterial metabolism and lipolysis, with bacterial injection markedly accelerating the pace of these metabolic processes.

VOCs fingerprint profiles were generated using the Gallery Plot plug-in for HS-GC-IMS 48 h post-inoculation. A dynamic progression was observed in the fluctuations of group M (Figure 1C) and group L (Figure 1D), which were characterized by an initial increase in chemical quantity followed by a progressive decline. In group M, changes in VOC composition and signal intensity became apparent from 4 h after inoculation onward. Conversely, the volatility profile of group L exhibited remarkable stability following the alteration at 4 h after inoculation, and the VOC concentration did not exhibit substantial variation from 4 to 8 h. The disparities in VOCs signatures between the two groups were primarily focused on the 12 h and 24 h time points, indicating temporal variations in metabolite composition.

#### 3.1.1. Alcohols

Six alcohols (3-methylbutanol M, 3-methylbutanol D, 2-methylpropanol, 1-octen-3-ol, 1-hexanol, and 3-methyl-3-buten-1-ol) were identified in group M, while ten alcohols (3-methylbutanol M, 3-methylbutanol D, 1-octen-3-ol, 1-pentanol M, 2-propanol, 1-butanol, 1-pentanol D, and 3-methyl-3-buten-1-ol) were identified in group L. Among the co-detected alcohols, 3-methylbutanol M and 3-methylbutanol D were more pronounced at 8–12 h, whereas 1-octen-3-ol and 3-methyl-3-buten-1-ol showed greater abundance at 24–48 h (Figure 1C).

Among the detected alcohols, several compounds exhibited similar variation patterns during contamination. 3-Methylbutanol, 1-propanol, and 2-propanol showed a trend of initial increase followed by a decrease. These alcohols are mainly derived from amino acid catabolism and the reduction of corresponding aldehydes, and their dynamic changes may reflect the balance between microbial production and subsequent metabolic consumption [13].

Branched-chain alcohols, particularly 3-methylbutanol and 2-methylpropanol, are closely associated with amino acid-derived microbial metabolism. These compounds can be generated through Ehrlich-like pathways, in which branched-chain amino acids are first converted into α-keto acids via aminotransferase-mediated reactions, followed by decarboxylation to aldehydes and subsequent reduction to higher alcohols by alcohol dehydrogenases [20,21]. In *E. coli*, leucine- and valine-derived intermediates have been demonstrated to serve as precursors for branched-chain alcohol formation, including 3-methylbutanol, through α-keto acid conversion pathways [22]. Therefore, the increase of 3-methylbutanol observed during early sampling stage may be related to amino acid catabolism occurring under the present inoculation conditions.

In contrast, 1-octen-3-ol and 2-hexanol showed a gradual accumulation pattern, with peak levels observed at later sampling stages. 1-Octen-3-ol is mainly generated from polyunsaturated fatty acids through lipid oxidation [23], and its formation may involve lipoxygenase-mediated oxidation of fatty acid precursors such as eicosapentaenoic acid and arachidonic acid [24]. The increased abundance of 1-octen-3-ol after 24 h may be associated with lipid oxidation during the later sampling stages. 1-Butanol, 1-pentanol, 2-methylpropanol, and 1-hexanol exhibited early-stage accumulation followed by a decline during storage. These compounds are mainly formed through lipid degradation, particularly via the breakdown of peroxyl or alkoxyl radicals, and have been reported in various meat systems, including beef, pork, and poultry. The decrease in their abundance over time may be associated with further oxidation or microbial utilization. Specifically, 1-pentanol, a product of polyunsaturated fatty acid oxidation, showed a continuous decrease with prolonged contamination, indicating its involvement in microbial degradation processes. In addition, 1-propanol has been reported as a potential marker associated with *E. coli* contamination under specific experimental conditions [25], whereas 2-propanol has been rarely detected in animal-derived foods. The temporal variation observed for these compounds may reflect the combined contribution of microbial activity, lipid-derived reactions, and other biochemical processes occurring during incubation.

#### 3.1.2. Aldehydes and Ketones

Aldehydes and ketones are important VOCs associated with flavor changes and rancidity in foods and are mainly derived from lipid oxidation and amino acid metabolism. During lipid oxidation, polyunsaturated fatty acids undergo free radical reactions to form hydroperoxide intermediates, which further decompose into characteristic aldehydes and ketones. Ketones may also originate from Strecker degradation, the Maillard reaction, lipid hydrolysis, alkane degradation, and alcohol dehydrogenation by Gram-negative bacteria [26]. In meat products, the degradation of proteins and peptides further increases the pool of free amino acids, thereby promoting the formation of fatty, aromatic, and sulfur-containing aldehydes.

Four ketones were concurrently identified in the M and L groups. Cyclohexanone was identified between 4 and 12 h, peaking at 8 h before progressively declining. Within group M, 2-pentanone was detected at 0 h, increased to its highest abundance at 8 h, and subsequently decreased. Pentane-2,3-dione also showed temporal variation, while acetoin reached a higher abundance at the later sampling stage. Within group L, 2-pentanone showed its highest abundance at 4 h, whereas pentane-2,3-dione and acetoin also exhibited changes in abundance across sampling times. (Figure 1C, Tables S2 and S3).

Several ketones and aldehydes showed distinct temporal patterns during *E. coli* incubation, reflecting dynamic changes in lipid oxidation, amino acid catabolism, and microbial metabolism. Several compounds, including cyclohexanone, acetone, hydroxypropionaldehyde, and benzaldehyde, were mainly detected at the early sampling stages and subsequently declined. Acetone was detected in group L within the first 8 h and then gradually declined, which may be related to its conversion into 2-propanol through microbial reduction. Similarly, benzaldehyde was observed in both groups during 0–8 h and reached its highest abundance at 4 h, suggesting its association with early-stage VOC changes potentially related to Strecker degradation and aromatic compound metabolism.

In contrast, several compounds accumulated during the intermediate and later sampling stages, including 2-heptanone, phenylacetaldehyde, and 1-octen-3-ol-related oxidative products. 3-(Methylthio)propanal showed temporal variation during incubation, with its abundance changing primarily during the intermediate sampling period. This compound is commonly associated with amino acid-related reactions, although its specific formation mechanism in the present chicken matrix was not directly verified. This unstable sulfur-containing aldehyde has been reported to promote spoilage-related VOC accumulation and negatively affect meat flavor quality [27]. Branched-chain aldehydes, including 2-methylbutanal and 3-methylbutanal, were mainly concentrated during the early sampling stages in both groups and may originate from microbial amino acid catabolism. Their subsequent decline may be related to further oxidation into ketones or carboxylic acids. Overall, these dynamic changes indicate that ketones and aldehydes in inoculated chicken meat are closely associated with lipid oxidation, carbohydrate degradation, and amino acid metabolism, and their temporal distribution may provide useful information for tracking spoilage progression.

#### 3.1.3. Acids and Esters

Three acids were identified in both groups M and L. Among them, 2-methylpropionic acid was the predominant component, with its concentration reaching a maximum at 4 h before exhibiting a declining trend (Figure 1C,D). Methyl acetate and propyl acetate were detected in both groups during the intermediate sampling period (8–24 h). 2-Methylpropionic acid has been associated with microbial branched-chain amino acid metabolism. In microorganisms capable of this pathway, valine can first be transaminated to α-ketoisovalerate, followed by subsequent reactions leading to isobutyric acid formation [28].

Butanoic acid exhibited the highest signal intensity at 48 h in group M, indicating increased abundance at the later sampling stage. Across the sampling period, several alcohols showed decreasing abundance, whereas some acids tended to increase at later sampling points. Alcohols can be oxidized to produce acidic compounds. At 12 h, acetic acid and propionic acid identified in group L exhibited off-odor characteristics, including rancid and cheesy scents. Acetic acid can be produced during anaerobic carbohydrate metabolism by *E. coli*. [29], and has also been reported as a volatile indicator associated with spoilage in smoked salmon [30].

### 3.2. Identification of Characteristic Volatiles Using HS-SPME-GC-MS

**Figure 2:**
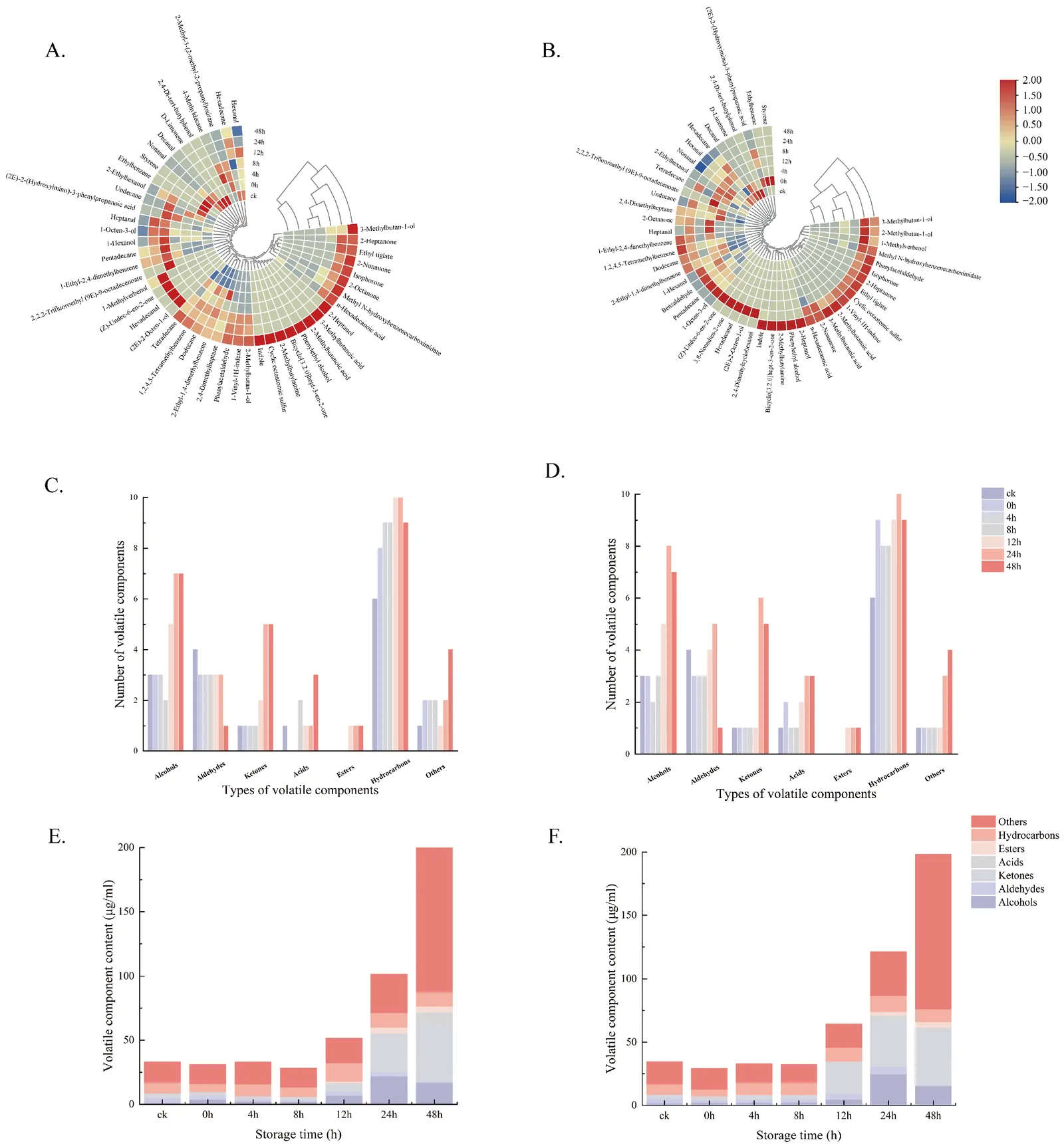
HS-SPME-GC-MS analysis of changes in volatile compounds in 48 h. (**A**) Clustering heatmap for the higher-load group; (**B**) Clustering heatmap for the lower-load group; (**C**) Changes in the number of compounds in the higher-load group; (**D**) Changes the number of compounds in the lower-load group; (**E**) Changes in compound concentrations in the higher-load group; (**F**) Changes in compound concentrations in the lower-load group.

HS-SPME-GC-MS exhibited varying sensitivity in the characterization of volatile compounds compared to the HS-GC-IMS technique. A total of 50 molecules were isolated via HS-SPME-GC-MS, comprising 10 alcohols, 7 aldehydes, 7 ketones, 4 acids, 2 esters, 14 hydrocarbons, 4 nitrogen-sulfur compounds, and 2 other compounds (Table S4). Minimal variation was observed in total volatile compositions between group M (46 species) and group L (48 species). The following compounds were consistently identified in both groups M and L: acids (4 species), esters (2 species), nitrogen-sulfur compounds (4 species), 9 of 10 alcohols, 6 of 7 aldehydes, 6 of 7 ketones, 13 of 14 hydrocarbons, and 1 of 2 additional compound. (Figure 2).

#### 3.2.1. Alcohols

Alcohols are mainly formed through microbial redox reactions related to lipid degradation and amino acid metabolism. Nine alcohols were collectively identified in groups M and L. Additionally, 2,4-dimethylcyclohexanol was detected just in group L (Figure 2A,B). 2-Heptanol and phenylethyl alcohol increased with incubation time, peaking at 48 h. (2E)-2-octen-1-ol and 1-methylverbenol were first detected at 24 h post-contamination and subsequently decreased at later sampling points. 2-Ethylhexanol was initially identified at 0 h following *E. coli* contamination decreased gradually, reappeared in minor amounts at 12 h, and then vanished by 48 h. Concentrations of 3-methylbutanol and 2-methylbutanol were detected from 12 h onward and showed increasing abundance at later sampling points. 1-Hexanol and 1-octen-3-ol also exhibited temporal variation, with their overall abundance tending to increase over the incubation period.

#### 3.2.2. Aldehydes and Ketones

Aldehydes can influence aroma, generating pleasant scents in meat products at low concentrations and rotten odors at elevated concentrations, potentially serving as indicators of aromatic rancidity in meat. Seven aldehydes and seven ketones were identified using HS-SPME-GC-MS. Hexanal, heptanal, phenylacetaldehyde, nonanal, decanal, and hexadecanal were collectively identified in both groups among the aldehydes. Hexanal, nonanal, and decanal showed a continuous decrease over time, whereas phenylacetaldehyde, heptanal, and hexadecanal presented an upward trend; nevertheless, the contents of heptanal and hexadecanal decreased at 48 h. Most of the identified ketones showed increasing abundance over the incubation period, which may be associated with lipid-related oxidative processes. 3,8-Nonadien-2-one was only identified in group L and exhibited a slow decline following its emergence at 24 h (Figure 2B).

Ketones are important volatile compounds in foods, mainly formed through lipid oxidation, amino acid metabolism, and microbial metabolism. However, due to their relatively high odor thresholds, they generally make a limited contribution to flavor development [31]. Several ketones, including 2-nonanone, 2-octanone, 2-heptanone, bicyclo[3.2.0]hept-3-en-2-one, and isophorone, were detected during incubation. Their formation may involve lipid oxidation, amino acid-related reactions, and other biochemical processes, although the specific pathways were not directly verified in the present chicken matrix [32]. The presence of ketones in later storage phases indicated that storage duration influenced VOCs in inoculated chicken tissue, likely associated with progressive biochemical degradation, lipid oxidation, and microbial metabolic activity during storage [33]. Ketone profiles may also be affected by multiple biological and matrix-related factors [34].

#### 3.2.3. Acids and Esters

The synthesis of acids and esters is linked to alcohols and free fatty acids, particularly the oxidation of alcohols and the generation of free fatty acids from long-chain fatty acids, which may result in potent scents. Four acids (3-methylbutanoic acid, 2-methylbutanoic acid, (2E)-2-(hydroxyimino)-3-phenylpropanoic acid, and n-hexadecanoic acid) were detected along with two esters: ethyl tiglate and 2,2,2-Trifluoroethyl (9E)-9-octadecenoate. 3-Methylbutanoic acid was commonly present in fermented food, contributing a cheesy, rotten, and sweaty aroma to these items [35]. 3-Methylbutanoic acid and 2-methylbutyric acid were synthesized in substantial amounts at 48 h, (Figure 2E,F, Tables S5 and S6), indicating a probable correlation between the concentrations of these acids and the microbial population. 3-Methylbutanoic acid can be associated with leucine catabolism, involving the formation of branched-chain intermediates that may subsequently be converted into the corresponding acid. Previous studies in fermented meat systems have reported the formation of 3-methylbutanol and 2-methylbutanol and their subsequent oxidation to the corresponding branched-chain acids, including 3-methylbutanoic acid and 2-methylbutanoic acid [35]. The concentrations of these compounds have been reported to vary with fat composition and the inoculation of fermenting bacteria, while these branched-chain alcohols can be subsequently oxidized to form substantial amounts of 2-methylbutyric and 3-methylbutyric acids [36]. N-Hexadecanoic acid is an important metabolite involved in lipid metabolism and has been reported to be associated with meat spoilage [37]. Esters are mainly generated through esterification reactions between alcohols and acids and are important contributors to odor formation. In the present study, both esters showed increasing abundance over the incubation period, although their specific formation pathways in the present chicken matrix were not directly verified.

#### 3.2.4. Hydrocarbons

The oxidative degradation of fatty acids often generates hydrocarbons and are generally regarded as having no impact on meat flavor due to their elevated odor threshold. The primary hydrocarbons in fermented beef products are predominantly aliphatic and aromatic. Fourteen hydrocarbons were identified: eight aliphatic hydrocarbons and six aromatic molecules. In the present study, alkane levels generally increased with contamination time (Figure 2C–F), indicating further fatty acid oxidation. In contrast, aliphatic hydrocarbons exhibited time-dependent variation patterns during contamination. Most alkanes, including undecane, pentadecane, tetradecane, and hexadecane, showed an initial increase followed by a decline, reflecting dynamic lipid degradation processes. Among them, undecane showed a decreasing trend at later sampling points, whereas tetradecane remained detectable at 48 h. Dodecane showed a similar fluctuating pattern, with initial detection at 4 h and accumulation at later sampling stages, while 4-methylheptane was uniquely detected in group M and exhibited relatively higher abundance. These hydrocarbons are mainly generated from the homolytic cleavage of alkoxy radicals derived from long-chain fatty acids during lipid degradation and may act as intermediates in the formation of aldehydes and ketones.

Six aromatic hydrocarbons were detected, exhibiting an increase in abundance after 24 h relative to aliphatic hydrocarbons. Ethylbenzene and styrene were detected at 0 h and gradually decreased at subsequent sampling points, indicating that they were mainly associated with the early temporal VOC profile under the present experimental conditions.

#### 3.2.5. Nitrogen, Sulfur and Other Compounds

Three nitrogenous compounds (2-methylbutylamine, methyl N-hydroxybenzenecarboximidate, indole) and one sulfide (cyclic octatomic sulfur) were identified (Figure 2A,B). Bacteria produce biogenic amines in meat by decarboxylating specific amino acids with substrate-specific decarboxylases [38], which explains why different microorganisms produce different volatile compounds. Volatile sulfur molecules are accountable for the sulfurous and rancid aromas primarily emitted by stored meat, most of which arise from the decomposition of sulfur-containing amino acids. At 48 h, 2-methylbutylamine, indole, and cyclic octatomic sulfur were detected, suggesting the development of more pronounced spoilage-related VOC characteristics and off-odors. Two compounds (2-methyl-3-(2-methyl-2-propanyl) oxirane and 2,4-Di-tert-butylphenol) were identified. 2-Methyl-3-(2-methyl-2-propanyl) oxirane was observed exclusively during 0–8 h in group M and then vanished. 2,4-Di-tert-butylphenol was detected in both inoculated groups and in CK, suggesting that it may be associated with the chicken matrix rather than specifically with *E. coli* inoculation.

### 3.3. Analysis of VOC Changes Between Two Methods Using PCA and OPLS-DA

**Figure 3:**
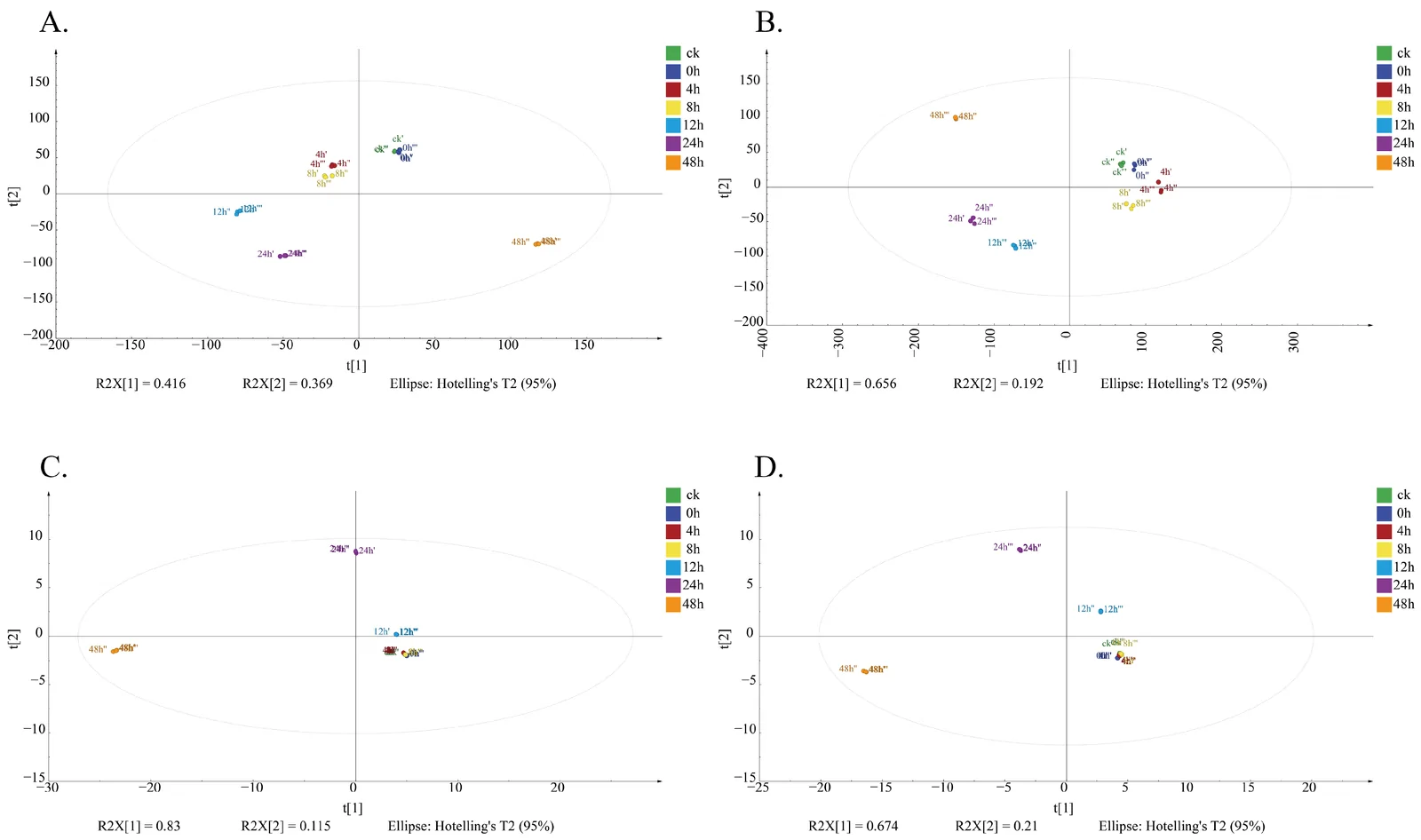
Principal component analysis score scatter plots among different incubation times. (**A**) Higher-load group by HS-GC-IMS; (**B**) Lower-load group by HS-GC-IMS; (**C**) Higher-load group by HS-SPME-GC-MS; (**D**) Lower-load group by HS-SPME-GC-MS.

VOCs in *E. coli*-inoculated chicken meat were characterized using HS-SPME-GC-MS and HS-GC-IMS. The results indicated that HS-GC-IMS detected a greater number of VOCs than HS-SPME-GC-MS and provided a clearer characterization of their dynamic changes. PCA and OPLS-DA were further used to visualize and characterize variations in the overall VOC profiles across sampling times within each initial inoculation group. The PCA score plots showed time-related distribution patterns of VOC profiles within groups M and L. (Figure 3). PCA based on HS-SPME-GC-MS and HS-GC-IMS data showed clear temporal separation of VOC profiles during *E. coli* contamination. For HS-SPME-GC-MS, the cumulative variance explained in group M and group L reached 94.5% and 88.4%, respectively, with time-related distribution patterns observed within each group along PC1. In contrast, HS-GC-IMS provided a clearer visualization of the distribution of early-stage samples (0–8 h), with cumulative variances of 78.5% and 84.8% for groups M and L, respectively. No observations were located outside the 95% Hotelling’s T^2^ confidence ellipse in the PCA score plots. Overall, HS-SPME-GC-MS provided broader information on relatively higher-molecular-weight VOCs. whereas HS-GC-IMS provided complementary information on changes in relatively low-molecular-weight VOCs, particularly during the early sampling stages. These findings highlight the complementary value of the two techniques for characterizing temporal VOC profiles under the present experimental conditions.

**Figure 4:**
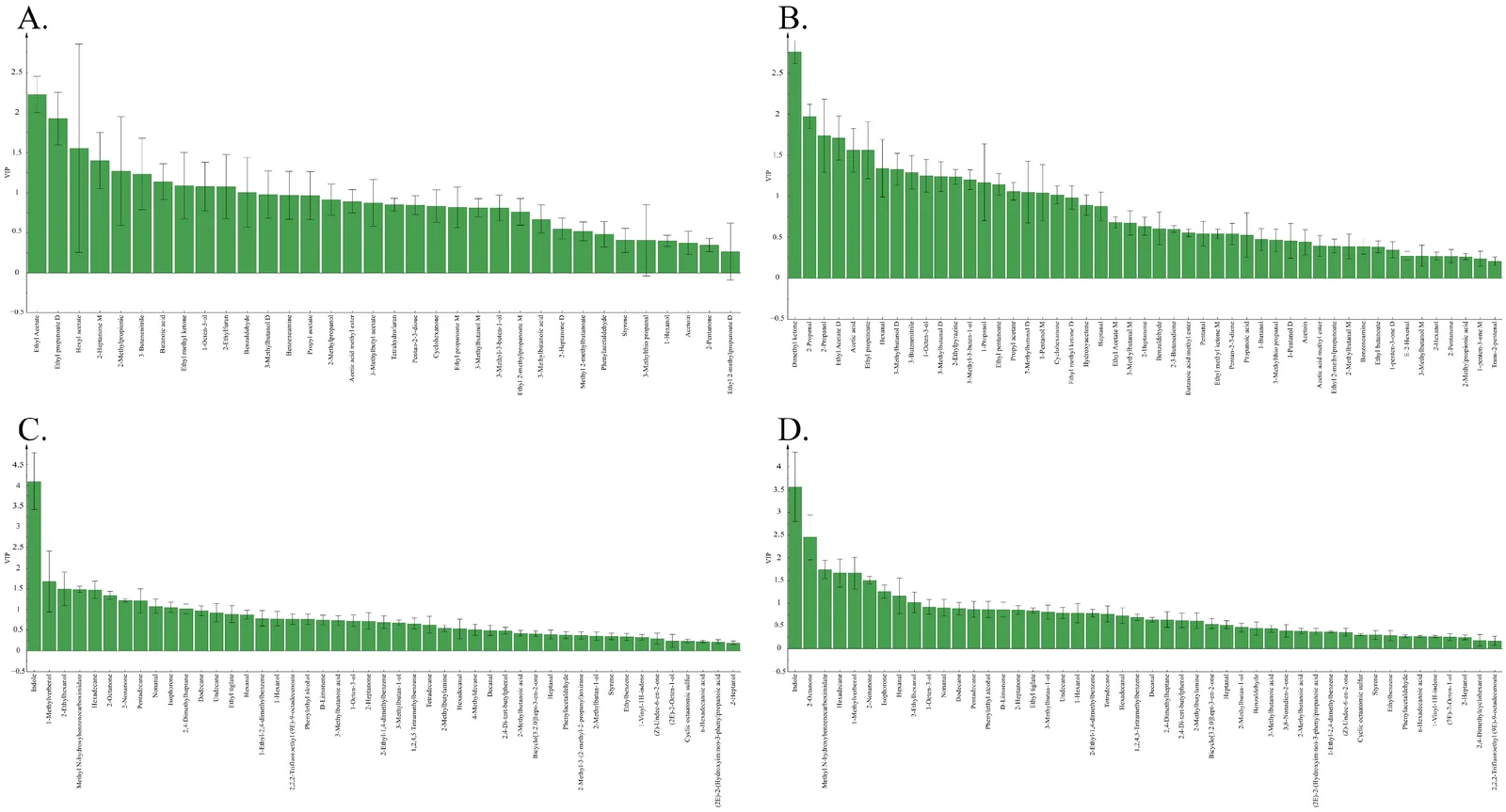
The variable importance in projection (VIP) scores of volatile compounds. (**A**) VIP diagram of higher-load group by HS-GC-IMS; (**B**) VIP diagram of the lower-load group by HS-GC-IMS; (**C**) VIP diagram of higher-load group by HS-SPME-GC-MS; (**D**) VIP diagram of the lower-load group by HS-SPME-GC-MS.

OPLS-DA was performed to visualize and characterize time-related variation in VOC profiles within each initial inoculation group and to identify VOCs contributing to the observed separation among sampling times. The score plots and biplots (Figure S1) showed distinct distribution patterns among samples collected at different time points within groups M and L., consistent with the PCA results. For HS-SPME-GC-MS, the model showed excellent explanatory and predictive ability, the R^2^X, R^2^Y, and Q^2^ values were 0.999, 0.997, and 0.994 for group M, and 0.998, 0.998, and 0.997 for group L, respectively. For HS-GC-IMS, the corresponding values were 0.997, 0.984, and 0.934 for group M, and 0.994, 0.979, and 0.938 for group L. These high R^2^Y and Q^2^ values indicated a strong fit and cross-validated performance of the models within the present dataset. The 200-permutation tests were further used to assess potential model overfitting. (Figure S2), and the permuted Q^2^ values were lower than those of the corresponding original models, supporting the validity of the observed model separation under the present dataset. To further identify VOC variables contributing to the time-related separation within each inoculation group, VIP analysis was performed based on the OPLS-DA model (Figure 4). Compounds with VIP > 1 were considered important contributors to the corresponding OPLS-DA models [6]. In HS-SPME-GC-MS, 11 and 8 VOCs with VIP > 1 were identified in groups M and L, respectively, whereas 10 and 19 compounds were identified in the corresponding HS-GC-IMS models.

**Figure 5:**
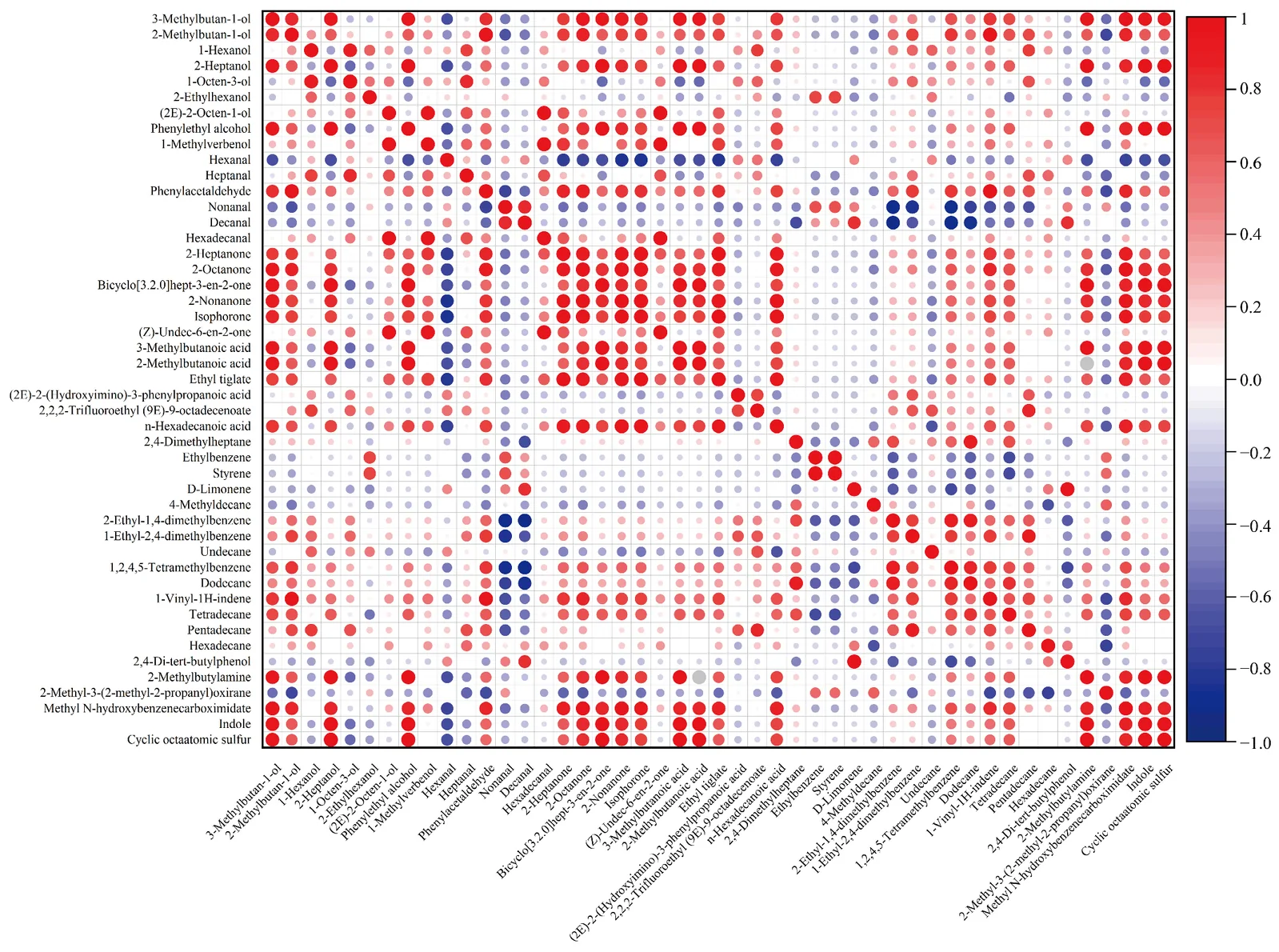
Correlation analysis of volatile compounds detected by HS-SPME-GC-MS. The color indicates the direction and strength of the correlation coefficient, with red and blue representing positive and negative correlations, respectively. The size of the circles represents the absolute value of the correlation coefficient, with larger circles indicating stronger correlations.

The VOC correlation matrix showed patterns of positive and negative associations among the detected compounds. (Figure 5). Positive correlations were observed among several aldehydes, ketones, and alcohols, reflecting similarities in their abundance variation across the analyzed samples. Branched-chain alcohols and acids also showed positive correlations, suggesting coordinated variation among these compounds under the present experimental conditions. In contrast, some long-chain hydrocarbons showed negative correlations with other VOCs, indicating different patterns of abundance variation. These correlations describe co-variation among VOCs and were not interpreted as direct evidence of common metabolic pathways or metabolic interactions.

### 3.4. Effects of Detection Methods and Initial Inoculum Levels on VOC Variation

**Figure 6:**
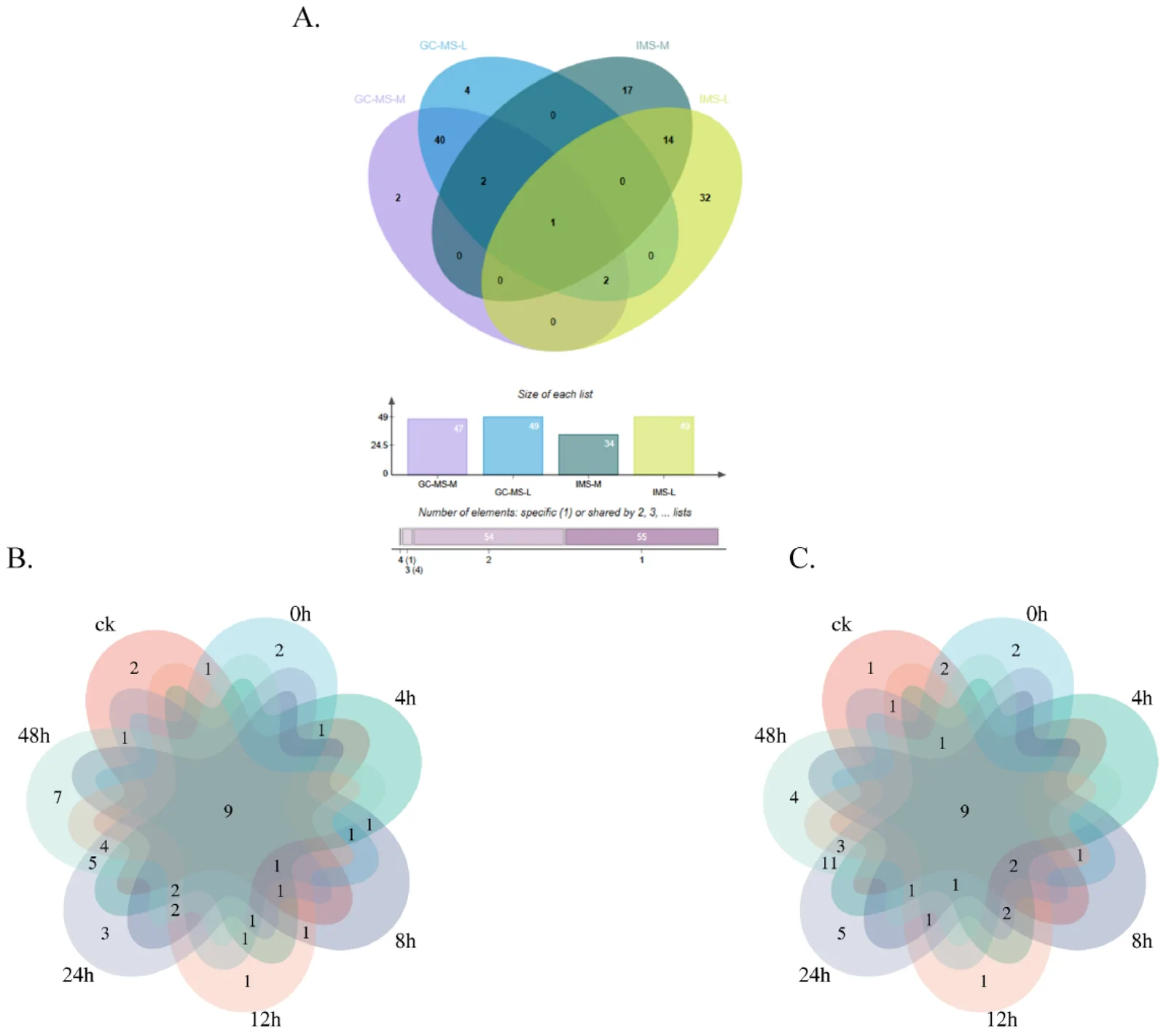
Venn diagram. (**A**) Venn diagram based on different detection methods. (**B**) Distribution of shared and time-specific VOCs detected by HS-SPME-GC-MS in the higher-load group across the seven sampling points (CK, 0, 4, 8, 12, 24, and 48 h). (**C**) Distribution of shared and time-specific VOCs detected by HS-SPME-GC-MS in the lower-load group across the same sampling points. Numbers in the overlapping and non-overlapping regions indicate the numbers of shared and time-specific VOCs, respectively.

Among the two detection techniques performed in this study, more compounds were detected by HS-GC-IMS (57 compounds) than by HS-SPME-GC-MS (50 compounds) (Figure 6B,C). HS-GC-IMS detected a greater number of small-molecule VOCs, especially alcohols, aldehydes, and ketones, and identified more compounds in these categories (Tables S1 and S4). In addition, its relatively rapid analytical workflow supports its potential application for rapid VOC fingerprint screening. By contrast, HS-SPME-GC-MS was more suitable for the identification and quantification of a broader range of volatile compounds in complex food matrices, particularly compounds with low proton affinity that are difficult to detect by IMS [39]. Therefore, the combined use of HS-GC-IMS and HS-SPME-GC-MS enabled more comprehensive characterization of VOC profiles.

1-Octen-3-ol was detected by both methods and showed a gradual increase in abundance during the later sampling stages of contamination (24–48 h). It is commonly associated with lipid oxidation in meat and may also be influenced by microbial activity. Previous studies have reported that 1-octen-3-ol is widely present in animal-derived foods and can be formed through the oxidation of unsaturated fatty acids, including linoleic acid [24]. Its abundance has also been reported to correlate with thiobarbituric acid reactive substances (TBARS), an indicator of lipid oxidation [40]. Therefore, 1-octen-3-ol has been reported as an important volatile compound associated with meat quality degradation.

Consistent with the temporal changes in VOCs described above, both analytical methods revealed time-related changes in VOC composition over the sampling period. Within the individual inoculation groups, fewer VOCs were observed at the early stage of contamination (0–12 h), whereas compound diversity increased over time. A decrease in the number of detected VOCs was observed in group L at 48 h, which may reflect further transformation or degradation of some volatile compounds. HS-GC-IMS predominantly detected aldehydes and ketones associated with lipid oxidation, amino acid metabolism, Strecker degradation, and Maillard reactions. Among these, cyclohexanone and acetoin were mapped to microbial metabolism in diverse environments (map01120), although their specific formation routes in the present chicken matrix were not experimentally verified. Several early-stage compounds, including benzaldehyde and 3-(methylthio) propanal, were detected at relatively higher levels at earlier sampling points and subsequently decreased after 12 h, reflecting temporal changes in their abundance during incubation. In contrast, 3-methylbutanol accumulated markedly at later sampling stages and was closely associated with microbial activity. 3-Methylbutanol has previously been detected in the volatile profiles of *E. coli* culture [16]. However, the metabolic origin of 3-methyl-3-buten-1-ol in the present chicken matrix remains unclear. Previous studies on engineered *E. coli* have associated 3-methyl-3-buten-1-ol production with isoprenoid precursor pathways rather than demonstrating its direct formation from 3-methyl-1-butanol [41]. Therefore, its formation pathway under the present inoculation conditions requires further verification. In addition, compared with HS-GC-IMS, HS-SPME-GC-MS more frequently detected hydrocarbons and nitrogen and sulfur-containing compounds, providing complementary coverage of the VOC profile.

Differences in the number of detected VOCs were also observed between the two initial inoculation groups. Under the present experimental conditions, both analytical methods detected a greater number of VOCs in group L than in group M. However, because the present statistical analysis was not designed to formally evaluate the main effect of inoculation level or its interaction with incubation time, this observation is interpreted descriptively. Differences in substrate availability, oxidation processes, and metabolite turnover may potentially contribute to the observed VOC patterns, but these mechanisms were not directly investigated in the present study and require further verification.

### 3.5. Identification of VOC Markers and Their Potential Biotransformation Pathways During E. coli Contamination

Bacterial metabolites vary markedly across different stages of contamination. During the early sampling stage, bacteria mainly produce ATP through glucose metabolism, and the resulting VOCs are primarily derived from glycolysis, the TCA cycle, and related side pathways. As contamination progresses, metabolism gradually shifts toward the accumulation of byproducts associated with energy metabolism and substrate degradation. In addition, fatty acids can be converted into acetyl-CoA via β-oxidation and further transformed by microbial activity, with some intermediates subsequently converted into alkanes through fatty acid decarboxylation. These changes may contribute to the dynamic evolution of VOC profiles during contamination.

**Figure 7:**
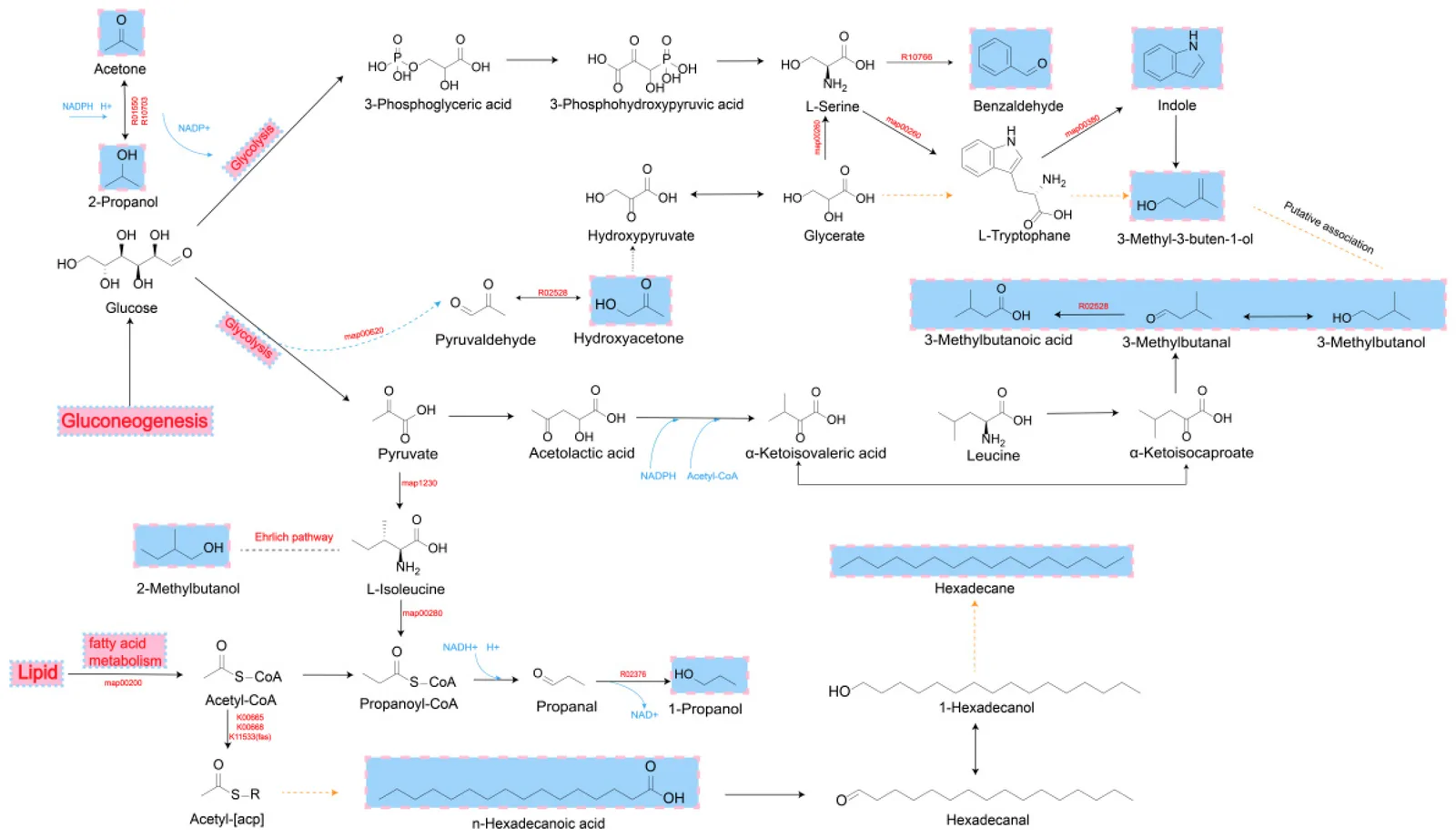
Putative metabolic pathways associated with VOC formation. Black arrows indicate general biochemical conversions, orange dashed arrows represent proposed VOC formation pathways, and blue labels indicate associated enzymes or cofactors involved in the reactions.

Based on HS-GC-IMS analysis, compounds unique to the blank control (CK) and those with VIP < 1 were excluded. Ethyl acetate, 3-butenenitrile, and 1-octen-3-ol showed positive correlations with time, suggesting that these VOCs may reflect time-dependent changes in *E. coli*-inoculated chicken meat. During the early sampling stage (0–8 h), VOCs were dominated by short-chain aldehydes, including hexanal, pentanal, and benzaldehyde. Hexanal and pentanal are mainly derived from the β-cleavage of polyunsaturated fatty acids such as linoleic acid, whereas benzaldehyde may originate from amino-acid-related reactions and other matrix-associated processes. As lipid peroxidation and microbial metabolism progressed, the abundance of these aldehydes decreased, accompanied by a gradual loss of fresh, grassy aroma and the emergence of spoilage-related odors. In the middle sampling stage (8–24 h), compounds derived from amino acid metabolism became more prominent. Among them, 3-(methylthio) propanal showed a transient increase between 8 and 12 h and may be associated with intermediate-stage changes in *E. coli*-inoculated chicken meat. Its formation is closely related to amino acid catabolism, and it has been reported to accelerate the accumulation of spoilage-related VOCs by inhibiting the formation of desirable meat aroma precursors [27]. In the same stage, 3-methylbutanol exhibited a sustained increase (4–24 h), suggesting its association with mid-stage changes following *E. coli* inoculation. It is mainly formed through microbial amino acid catabolism and the reduction of branched-chain aldehydes, involving the sequential conversion of amino acids (e.g., leucine, isoleucine, methionine, and phenylalanine) into α-keto acids, aldehydes, and corresponding alcohols [23]. Due to its close association with amino acid degradation, 3-methylbutanol is widely regarded as an indicator of microbial spoilage [42]. In the later sampling stages of spoilage (24–48 h), further transformation of these intermediates led to the accumulation of secondary alcohols and related compounds. 3-Methylbutanol may be further converted into 3-methyl-3-buten-1-ol, which showed a strong correlation with time and may be associated with late-stage changes in *E. coli*-inoculated chicken meat. In addition, 1-propanol and 2-propanol were enriched, indicating enhanced microbial metabolic activity. KEGG pathway mapping suggested 2-propanol to the reversible reduction of acetone within butanoate metabolism. However, this database-based association represents a possible biochemical relationship rather than direct evidence for the operation of this pathway in the inoculated chicken matrix. Therefore, the specific mfetabolic origin of 2-propanol under the present conditions remains to be verified (Figure 7).

Based on HS-SPME-GC-MS analysis, compounds unique to the CK group and those with VIP < 1 were excluded, and five volatile metabolites (indole, methyl N-hydroxybenzenecarboximidate, 2-octanone, 2-nonanone, and isophorone) were identified as potential discriminatory VOCs. Phenylacetaldehyde and hexadecanal increased markedly over time, reflecting enhanced amino acid metabolism and fatty acid degradation, respectively. Phenylacetaldehyde was mapped to phenylalanine metabolism pathway (map00360), and its accumulation has been reported to be closely associated with microbial activity, particularly involving *Enterobacteriaceae* and *Staphylococcus* [43]. In addition, 3-methylbutyric acid, potentially associated with leucine degradation, and indole, a product related to tryptophan catabolism, also showed increased abundance during the later sampling stages. These late-stage VOCs are closely associated with intensified microbial metabolism, particularly amino acid catabolism and lipid degradation. Spoilage-associated bacteria, such as Pseudomonas and Enterobacteriaceae, can hydrolyze triglycerides by secreting lipases and proteases, releasing free fatty acids and amino acids and thereby promoting the formation of secondary alcohols [26].

Among amino acid-derived metabolites, phenethyl alcohol and 3-methylbutan-1-ol showed increased abundance during the later sampling stages. Phenethyl alcohol is produced via microbial conversion of phenylalanine, likely through the reduction of phenylacetaldehyde [43]. Similarly, 3-methylbutan-1-ol is mainly generated through microbial degradation of branched-chain amino acids via the Ehrlich pathway, involving sequential deamination, decarboxylation, and reduction reactions. In parallel, lipid degradation also contributed to VOC formation. Long-chain aldehydes such as hexadecanal, mapped to the fatty acid degradation pathway (map00071), may act as important intermediates that can be further converted into low-molecular-weight volatile compounds (Figure 7).

**Figure 8:**
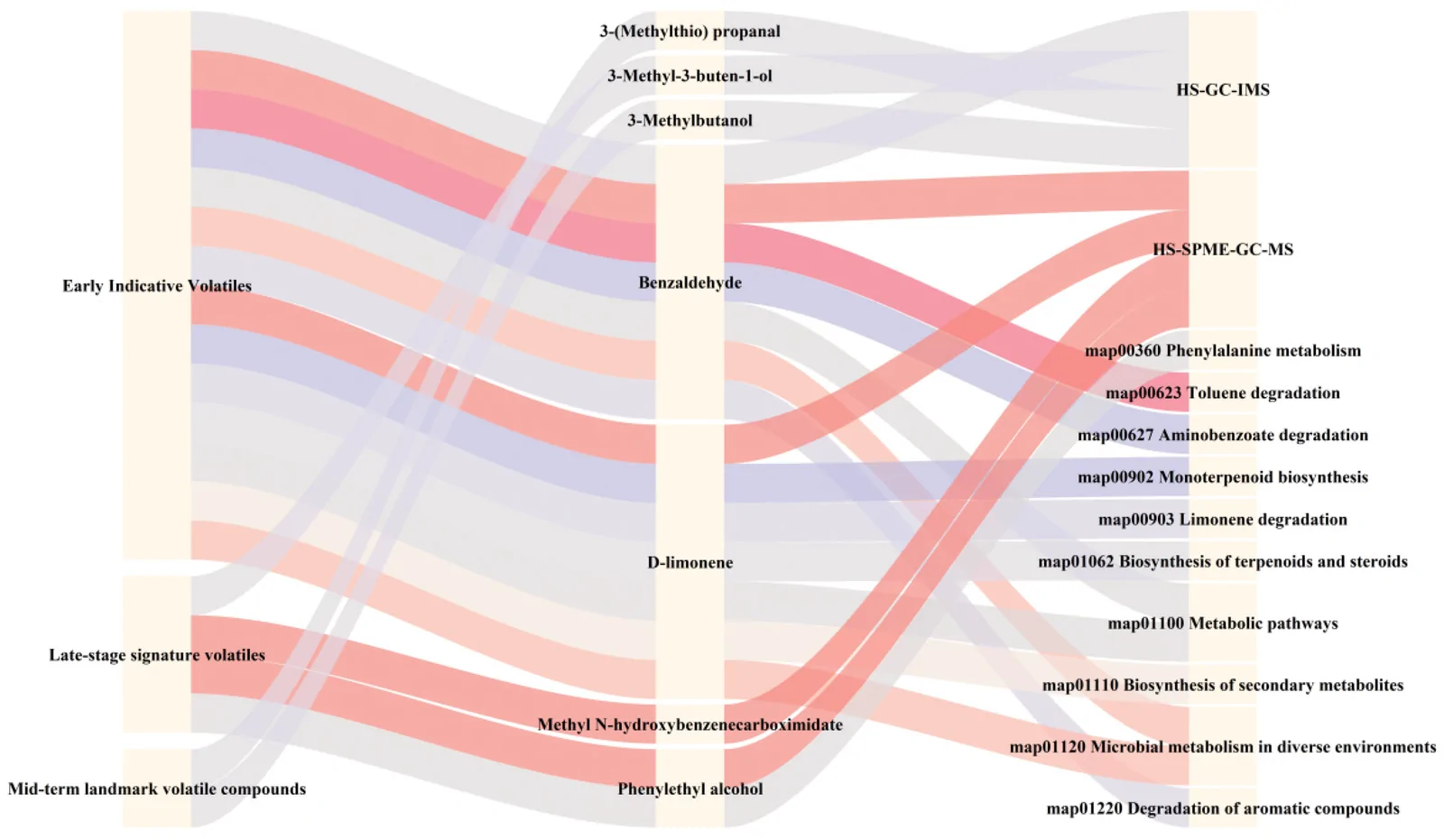
Signature volatile compounds and the pathway Sankey diagram. Different colors represent different temporal categories of signature volatile compounds, including early indicative volatiles, mid-term landmark volatile compounds, and late-stage signature volatile compounds. The width of the Sankey flows represents the association between volatile compounds and their corresponding analytical platforms or putative KEGG metabolic pathways.

Overall, the accumulation of VOCs at later sampling stages may reflect the combined contributions of amino acid catabolism, lipid degradation, and other biochemical processes occurring during incubation. Although some compounds exhibited low predictive contribution in the model (VIP < 1), their concentration trends remained consistent across groups M and L, and they were therefore retained as potentially relevant VOCs. The Sankey diagram further summarizes the identified VOCs by grouping them into early-, middle-, and late-stage-associated VOCs and linking them to their corresponding putative KEGG metabolic pathways (Figure 8).

## 4. Conclusions

In summary, HS-GC-IMS detected more low-molecular-weight VOCs during the early sampling stages, whereas HS-SPME-GC-MS provided complementary information on VOCs with relatively higher molecular weights and more complex formation pathways. The complementary use of these two analytical techniques enabled a more comprehensive characterization of the temporal VOC profiles in *E. coli*-inoculated chicken meat. Through HS-GC-IMS technology, benzaldehyde, 3-methylbutanol, 3-(methylthio) propanal, and 3-methyl-3-buten-1-ol were identified as VOCs associated with stage-dependent changes under the present *E. coli* inoculation conditions. HS-SPME-GC-MS analysis indicated phenethyl alcohol, and Methyl N-hydroxybenzenecarboximidate as candidate spoilage-associated compounds. The fluctuating variations in VOC profiles suggested that microbial activity was associated with ongoing biochemical transformations in chicken meat, encompassing lipid breakdown, amino acid metabolism, and the synthesis of alcohols, aldehydes, esters, hydrocarbons, nitrogenous, and sulphurous compounds. These results provide insights into VOC evolution during *E. coli*-associated contamination and underscore the promise of VOC-based methodologies for tracking alterations in microbial quality.

Nonetheless, several limitations should be considered. Only one *E. coli* strain was evaluated, and the lack of time-matched uninoculated controls and microbial growth data limits the specific attribution of temporal VOC changes to *E. coli*. In addition, the replication structure limited biological independence, and the statistical analyses did not formally evaluate the effect of initial inoculation level or its interaction with incubation time. Pre-analytical handling also differed across sampling times: rinsates collected at 0, 4, and 8 h were temporarily stored at −20 °C, whereas later samples were analyzed without freezing; therefore, an influence of sample handling on the observed temporal VOC patterns cannot be excluded. Finally, the proposed metabolic pathways were based on putative pathway mapping and require further biochemical validation. Accordingly, the identified VOCs should be regarded as candidate indicators associated with *E. coli* inoculation under the present conditions rather than *E. coli*-specific biomarkers.

Future studies should validate these candidate VOCs using time-matched uninoculated controls, naturally contaminated chicken samples, and realistic commercial storage conditions. Integrating such VOC signatures with rapid sensing technologies may further facilitate the development of practical tools for real-time food safety monitoring.

## Figures and Tables

**Table 1:** Comparison of representative VOC-based analytical approaches for chicken quality and microbial contamination assessment.

**Reference**	**Sample/Analytical Application**	**Analytical Technique**	**Main Advantages**	**Main Limitations/Comparison**
Zhong et al. [8]	Volatile flavor characterization of Hericium erinaceus during postharvest storage	E-nose + HS-GC-IMS + HS-SPME-GC-MS	E-nose enables rapid evaluation and visualization of overall VOC fingerprint changes	E-nose alone provides limited information for accurate identification of individual volatile compounds
Ning et al. [5]	Identification of key off-flavor compounds in chicken meat	HS-SPME + GC × GC-MS + GC-O-MS	Combines structural identification with direct sensory relevance of individual VOCs	More complex analytical workflow; GC-O involves sensory assessment and is less suitable for rapid routine screening
Wang et al. [12]	Early *S. aureus* contamination of raw chicken within 48 h	HS-SPME-GC-MS + HS-GC-IMS	Demonstrated strong complementarity between the two platforms; HS-GC-IMS was particularly sensitive to short-chain volatile compounds	Dual-instrument workflow increases analytical complexity; applicability of identified VOCs to other poultry products requires further validation
Wang et al. [13]	Early *Salmonella* contamination of chicken within 48 h	HS-SPME-GC-MS + HS-GC-IMS	Strong sensitivity to early contamination-related VOC changes and broad complementary VOC coverage	VOC profiles and candidate markers are pathogen- and matrix-dependent and therefore cannot be directly extrapolated to *E. coli* contamination
Present study	*E. coli*-contaminated chicken during 0–48 h storage	HS-SPME-GC-MS + HS-GC-IMS	Extends complementary dual-platform VOC analysis to *E. coli*-contaminated chicken; combines compound identification with sensitive VOC fingerprinting	Requires two analytical platforms; candidate VOC indicators require further validation under natural contamination and commercial storage conditions

## Data Availability

The data presented in this study are available within the article and its Supplementary Materials. Additional data are available on request from the corresponding author.

## Supplementary Materials

The following supporting information can be downloaded at https://www.mdpi.com/article/10.3390/foods15183198/s1. (Figure S1) Orthogonal partial least squares-discriminant biplots. (A) OPLS-DA biplot of high inoculation group by HS-GC-IMS; (B) OPLS-DA biplot of low inoculation group by HS-GC-IMS; (C) OPLS-DA biplot of high inoculation group by HS-SPME-GC-MS; (D) OPLS-DA biplot of low inoculation group by HS-SPME-GC-MS; (Figure S2) Permutation analysis. (A) Permutation analysis of high inoculation group by HS-GC-IMS; (B) Permutation analysis of low inoculation group by HS-GC-IMS; (C) Permutation analysis of high inoculation group by HS-SPME-GC-MS; (D) Permutation analysis of low inoculation group by HS-SPME-GC-MS; (Table S1) List of VOCs identified using HS-GC-IMS in E. coli-inoculated chicken meat stored for 0–48 h; (Table S2) Signal intensities of volatile compounds detected by HS-GC-IMS in the high inoculation group; (Table S3) Signal intensities of volatile compounds detected by HS-GC-IMS in the low inoculation group; (Table S4) List of VOCs identified using HS-SPME-GC-MS in *E. coli*-inoculated chicken meat stored for 0–48 h; (Table S5) Changes in volatile compounds during chicken meat storage (mean ± SD) and correlation analysis with storage time in the high inoculation level group of *E. coli*; (Table S6) Changes in volatile compounds during chicken meat storage (mean ± SD) and correlation analysis with storage time in the low inoculation level group of *E. coli*.

## Abbreviations

E-nose, electronic nose technology; GC-O-MS, gas chromatography–olfactometry–mass spectrometry; HS-SPME-GC-MS, headspace solid-phase microextraction-gas chromatography–mass spectrometry; GC-IMS, gas chromatography–ion migration spectroscopy; Group M, 10^−4^ bacterial suspension diluted with physiological saline; Group L, 10^−6^ bacterial suspension diluted with physiological saline; LRI, linear retention index; RI, retention index; NIST, the National Institute of Standards and Technology; PCA, principal component analysis; OPLS-DA, orthogonal partial least squares-discriminant analysis; VIP, variable importance in projection; 222 VOC, volatile organic compound; CK, chicken samples without *E. coli* for 0 h; SD, standard deviation; KEGG, kyoto encyclopedia of genes and genomes; ANOVA, analysis of variance; RIP, reactive ion peak; TBARS, thiobarbituric acid reactive substances; TCA, tricarboxylic acid cycle.

## References

[1] Jia Z., Luo Y., Wang D., Holliday E., Sharma A., Green M.M., Roche M.R., Thompson-Witrick K., Flock G., Pearlstein A.J. (2024). Surveillance of Pathogenic Bacteria on a Food Matrix Using Machine-Learning-Enabled Paper Chromogenic Arrays. Biosens. Bioelectron..

[2] OECD, Food and Agriculture Organization of the United Nations (2017). OECD-FAO Agricultural Outlook 2017–2026.

[3] Ju X., Zhang M., Shan Y., Liu Y., Tu Y., Ji G., Shu J. (2025). A Comprehensive Analysis of Meat Quality Degradation and Identification of Spoilage Markers in Chicken during Refrigerated Storage Using Multi-Method Approach. Food Chem..

[4] Azimzadeh M., Khashayar P., Mousazadeh M., Daneshpour M., Rostami M., Goodlett D.R., Manji K., Fardindoost S., Akbari M., Hoorfar M. (2025). Volatile Organic Compounds (VOCs) Detection for the Identification of Bacterial Infections in Clinical Wound Samples. Talanta.

[5] Ning H., Yang A., Zhou W., Li S., Tang Y., Ma W., Liu P. (2025). Key Volatile Off-Flavor Compounds Identification in Chicken Meat and Deodorizing Effects of Polyphenol and Spice Extracts. Food Chem. X.

[6] Zhou Y., Wang D., Zhao J., Guo Y., Yan W. (2025). Differentiation and Characterization of Volatile Compounds in Five Common Milk Powders Using HS-GC-IMS, HS-SPME-GC–MS, and Multivariate Statistical Approaches. Food Chem. X.

[7] Jia W., Liang G., Tian H., Sun J., Wan C. (2019). Electronic Nose-Based Technique for Rapid Detection and Recognition of Moldy Apples. Sensors.

[8] Zhong Y., Cui Y., Yu J., Yan S., Bai J., Xu H., Li M. (2024). Volatile Flavor Behavior Characterization of Hericium Erinaceus during Postharvest Storage Using E-Nose, HS-GC-IMS, and HS-SPME-GC–MS after Treated with Electron-Beam Generated X-Ray Irradiation. Food Chem..

[9] Li M., Tan H., Yang L., Zhou Y., Tan H., Lei H., Wei X. (2025). Characterization of Odor Skeleton Components and Prediction of Quality Grade in Qingyuan Chicken Based on HS-SPME-GC–MS. Food Chem..

[10] Zhao D., Shi D., Sun J., Li A., Sun B., Zhao M., Chen F., Sun X., Li H., Huang M. (2018). Characterization of Key Aroma Compounds in Gujinggong Chinese Baijiu by Gas Chromatography–Olfactometry, Quantitative Measurements, and Sensory Evaluation. Food Res. Int..

[11] Li Y., Yuan L., Liu H., Liu H., Zhou Y., Li M., Gao R. (2023). Analysis of the Changes of Volatile Flavor Compounds in a Traditional Chinese Shrimp Paste during Fermentation Based on Electronic Nose, SPME-GC-MS and HS-GC-IMS. Food Sci. Hum. Wellness.

[12] Wang Y., Wang X., Huang Y., Yue T., Cao W. (2023). Analysis of Volatile Markers and Their Biotransformation in Raw Chicken during Staphylococcus aureus Early Contamination. Foods.

[13] Wang Y., Wang X., Huang Y., Liu C., Yue T., Cao W. (2024). Identification and Biotransformation Analysis of Volatile Markers during the Early Stage of Salmonella Contamination in Chicken. Food Chem..

[14] Boots A.W., Smolinska A., Van Berkel J.J.B.N., Fijten R.R.R., Stobberingh E.E., Boumans M.L.L., Moonen E.J., Wouters E.F.M., Dallinga J.W., Van Schooten F.J. (2014). Identification of Microorganisms Based on Headspace Analysis of Volatile Organic Compounds by Gas Chromatography–Mass Spectrometry. J. Breath Res..

[15] Tait E., Perry J.D., Stanforth S.P., Dean J.R. (2014). Identification of Volatile Organic Compounds Produced by Bacteria Using HS-SPME-GC–MS. J. Chromatogr. Sci..

[16] Żuchowska K., Tracewska A., Depka-Radzikowska D., Bogiel T., Włodarski R., Bojko B., Filipiak W. (2025). Profiling of Volatile Metabolites of Escherichia Coli Using Gas Chromatography–Mass Spectrometry. Int. J. Mol. Sci..

[17] Wang J., Hang Y., Yan T., Liang J., Huang Z., Xu H. (2018). Qualitative Analysis of Flavors and Fragrances Added to Tea by Using GC–MS. J. Sep. Sci..

[18] Shi Q., Tang H., Mei Y., Chen J., Wang X., Liu B., Cai Y., Zhao N., Yang M., Li H. (2023). Effects of Endogenous Capsaicin Stress and Fermentation Time on the Microbial Succession and Flavor Compounds of Chili Paste (a Chinese Fermented Chili Pepper). Food Res. Int..

[19] Xiao N., Xu H., Jiang X., Sun T., Luo Y., Shi W. (2022). Evaluation of Aroma Characteristics in Grass Carp Mince as Affected by Different Washing Processes Using an E-Nose, HS-SPME-GC-MS, HS-GC-IMS, and Sensory Analysis. Food Res. Int..

[20] Hazelwood L.A., Daran J.-M., Van Maris A.J.A., Pronk J.T., Dickinson J.R. (2008). The Ehrlich Pathway for Fusel Alcohol Production: A Century of Research on *Saccharomyces cerevisiae* Metabolism. Appl. Environ. Microbiol..

[21] Yogiswara S., Verstrepen K.J. (2026). Recent Advances in Microbial 3-Methyl-1-Butanol Production. Front. Microbiol..

[22] Atsumi S., Hanai T., Liao J.C. (2008). Non-Fermentative Pathways for Synthesis of Branched-Chain Higher Alcohols as Biofuels. Nature.

[23] Acquaticci L., Angeloni S., Baldassarri C., Sagratini G., Vittori S., Torregiani E., Petrelli R., Caprioli G. (2024). A New HS-SPME-GC-MS Analytical Method to Identify and Quantify Compounds Responsible for Changes in the Volatile Profile in Five Types of Meat Products during Aerobic Storage at 4 °C. Food Res. Int..

[24] Song X., Canellas E., Nerin C. (2021). Screening of Volatile Decay Markers of Minced Pork by Headspace-Solid Phase Microextraction–Gas Chromatography–Mass Spectrometry and Chemometrics. Food Chem..

[25] Heng W.S., Jadhav S.R., Ueland M., Shellie R.A. (2023). Rapid Detection of Escherichia Coli in Dairy Milk Using Static Headspace-Comprehensive Two-Dimensional Gas Chromatography. Anal. Bioanal. Chem..

[26] Argyri A.A., Mallouchos A., Panagou E.Z., Nychas G.-J.E. (2015). The Dynamics of the HS/SPME–GC/MS as a Tool to Assess the Spoilage of Minced Beef Stored under Different Packaging and Temperature Conditions. Int. J. Food Microbiol..

[27] Liu J., Deng S., Wang J., Huang F., Han D., Xu Y., Yang P., Zhang C., Blecker C. (2024). Comparison and Elucidation of the Changes in the Key Odorants of Precooked Stewed Beef during Cooking-Refrigeration-Reheating. Food Chem. X.

[28] Leonardo I.C., Ferreira A., Bronze M.D.R., Quendera A.P., Fernandes A.F., Barreto Crespo M.T., Bustos Gaspar F. (2025). Beyond Guaiacol and Halophenols: Unravelling Isobutyric and Isovaleric Acids as New Culprits in off-Flavour Spoilage by *Alicyclobacillus* spp.. Int. J. Food Microbiol..

[29] Fitzgerald S., Duffy E., Holland L., Morrin A. (2020). Multi-Strain Volatile Profiling of Pathogenic and Commensal Cutaneous Bacteria. Sci. Rep..

[30] Iglesias J., Medina I. (2008). Solid-Phase Microextraction Method for the Determination of Volatile Compounds Associated to Oxidation of Fish Muscle. J. Chromatogr. A.

[31] Yu L., Pang Y., Shen G., Bai B., Yang Y., Zeng M. (2025). Identification and Selection of Volatile Compounds Derived from Lipid Oxidation as Indicators for Quality Deterioration of Frozen White Meat and Red Meat Using HS-SPME-GC–MS Combined with OPLS-DA. Food Chem..

[32] Lou X., Wen X., Chen L., Shu W., Wang Y., Hoang T.T., Yang H. (2023). Changes in Texture, Rheology and Volatile Compounds of Golden Pomfret Sticks Inoculated with Shewanella Baltica during Spoilage. Food Chem..

[33] Douglas S.L., Gilmore N.E., Barrazueta-Cordero R.J., Contreras X.M., Ball J.J., Mulvaney D.R., Rodning S.P., Sawyer J.T. (2025). Packaging Alters Fresh Chicken Characteristics and Volatile Profiles During Refrigerated Storage. Foods.

[34] Casaburi A., Piombino P., Nychas G.-J., Villani F., Ercolini D. (2015). Bacterial Populations and the Volatilome Associated to Meat Spoilage. Food Microbiol..

[35] Castada H.Z., Park C., Harper W.J., Barringer S.A. (2016). Suppression of Propanoic Acid, Acetic Acid and 3-Methylbutanoic Acid Production by Other Volatiles in a Swiss Cheese Curd Slurry System. Int. Dairy J..

[36] Corral S., Salvador A., Belloch C., Flores M. (2015). Improvement the Aroma of Reduced Fat and Salt Fermented Sausages by Debaromyces Hansenii Inoculation. Food Control..

[37] Yi Z., Xiao X., Cai W., Ding Z., Ma J., Lv W., Yang H., Xiao Y., Wang W. (2024). Unraveling the Spoilage Characteristics of Refrigerated Pork Using High-Throughput Sequencing Coupled with UHPLC-MS/MS-Based Non-Targeted Metabolomics. Food Chem..

[38] Alessandroni L., Caprioli G., Faiella F., Fiorini D., Galli R., Huang X., Marinelli G., Nzekoue F., Ricciutelli M., Scortichini S. (2022). A Shelf-Life Study for the Evaluation of a New Biopackaging to Preserve the Quality of Organic Chicken Meat. Food Chem..

[39] Yao W., Ma S., Wu H., Liu D., Liu J., Zhang M. (2024). Flavor Profile Analysis of Grilled Lamb Seasoned with Classic Salt, Chili Pepper, and Cumin (Cuminum Cyminum) through HS-SPME-GC-MS, HS-GC-IMS, E-Nose Techniques, and Sensory Evaluation on Sonit Sheep. Food Chem..

[40] Iglesias J., Medina I., Bianchi F., Careri M., Mangia A., Musci M. (2009). Study of the Volatile Compounds Useful for the Characterisation of Fresh and Frozen-Thawed Cultured Gilthead Sea Bream Fish by Solid-Phase Microextraction Gas Chromatography–Mass Spectrometry. Food Chem..

[41] Kim J., Lee T.S. (2023). Enhancing Isoprenol Production by Systematically Tuning Metabolic Pathways Using CRISPR Interference in *E. coli*. Front. Bioeng. Biotechnol..

[42] Yang C., Qi Y., Zheng J., Fan X., Liang P., Song C. (2018). Efficacy of Various Preservatives on Extending Shelf Life of Vacuum-Packaged Raw Pork during 4 °C Storage. J. Food Prot..

[43] Zang J., Yu D., Li T., Xu Y., Regenstein J.M., Xia W. (2022). Identification of Characteristic Flavor and Microorganisms Related to Flavor Formation in Fermented Common Carp (*Cyprinus carpio* L.). Food Res. Int..

