# Supplementary material for: Volatilomic Profiling of Early-Stage Biotransformation Enables Identification of Diagnostic Volatiles in Escherichia coli-Contaminated Chicken Meat
Source: Foods. 2026 Sep 10;15(18):3198. doi: 10.3390/foods15183198 (PMC13605107; doi:10.3390/foods15183198)
Supplement: Supplementary file 1 [file foods-15-03198-s001.zip › foods-4493781-supplementary.pdf]

Supporting Materials

Figure S1. Orthogonal partial least squares-discriminant biplots. (A) OPLS-DA biplot of high inoculation group by HS-GC-IMS; (B) OPLS-DA biplot of low inoculation group by HS-GC-IMS; (C) OPLS-DA biplot of high inoculation group by HS-SPME-GC-MS; (D) OPLS-DA biplot of low inoculation group by HS-SPME-GC-MS.

Figure S2. Permutation analysis. (A) Permutation analysis of high inoculation group by HS-GC-IMS; (B) Permutation analysis of low inoculation group by HS-GC-IMS; (C) Permutation analysis of high inoculation group by HS-SPME-GC-MS; (D) Permutation analysis of low inoculation group by HS-SPME-GC-MS.

Table S1. List of VOCs identified using HS-GC-IMS in *E. coli*-inoculated chicken meat stored for 0-48 h.

Table S2. Signal intensities of volatile compounds detected by HS-GC-IMS in the high inoculation group.

Table S3. Signal intensities of volatile compounds detected by HS-GC-IMS in the low inoculation group.

Table S4. List of VOCs identified using HS-SPME-GC-MS in *E. coli*-inoculated chicken meat stored for 0-48 h.

Table S5. Changes in volatile compounds during chicken meat storage (mean ± SD) and correlation analysis with storage time in the high inoculation level group of *E. coli.*

Table S6. Changes in volatile compounds during chicken meat storage (mean ± SD) and correlation analysis with storage time in the low inoculation level group of *E. coli.*

Table S1. List of VOCs identified using HS-GC-IMS in E. coli-inoculated chicken meat stored for 0-48 h.

Class	M	L	Compound	Formula	MW
Alcohols	+	+	3-methylbutanol M[2]	$C_5H_{12}O$	88.1
	+	+	3-methylbutanol D	$C_5H_{12}O$	88.1
	+	-	2-methylpropanol	$C_4H_{10}O$	74.1
	+	+	1-Octen-3-ol	$C_8H_{16}O$	128.2
	+	-	1-Hexanol	$C_6H_{14}O$	102.2
	-	+	1-Pentanol M[2]	$C_5H_{12}O$	88.1
	-	+	2-Propanol	C_3H_8O	60.1
	-	+	1-Butanol	$C_4H_{10}O$	74.1
	-	+	1-Pentanol D	$C_5H_{12}O$	88.1
	-	+	3-Methyl-3-buten-1-ol	$C_5H_{10}O$	86.1
	-	+	1-Propanol	C_3H_8O	60.1
	-	+	2-Hexanol	$C_6H_{14}O$	102.2
Aldehydes	+	+	Benzaldehyde	C_7H_6O	106.1
	+	-	phenylacetaldehyde	C_8H_8O	120.2
	-	+	2-methylbutanal M[2]	$C_5H_{10}O$	86.1
	-	+	2-methylbutanal D	$C_5H_{10}O$	86.1
	+	+	3-methylbutanal M[2]	$C_5H_{10}O$	86.1
	+	+	3-methylbutanal D	$C_5H_{10}O$	86.1
	-	+	Pentanal	$C_5H_{10}O$	86.1
	-	+	hexanal	$C_6H_{12}O$	100.2
	+	+	3-(methylthio) propanal	C_4H_8OS	104.2
	-	+	trans-2-pentenal	C_5H_8O	84.1
	-	+	Heptanal	$C_7H_{14}O$	114.2
	-	+	(E)-2-hexenal	$C_6H_{10}O$	98.1
Ketones	+	+	Cyclohexanone	$C_6H_{10}O$	98.1
	+	+	2-Heptanone M[2]	$C_7H_{14}O$	114.2

	+	+	2-Pentanone	$C_5H_{10}O$	86.1
	+	+	pentan-2,3-dione	$C_5H_8O_2$	100.1
	+	+	Acetoin	$C_4H_8O_2$	88.1
	+	+	2-Heptanone D	$C_7H_{14}O$	114.2
	-	+	Dimethyl ketone	C_3H_6O	58.1
	+	+	Ethyl methyl ketone M[2]	C_4H_8O	72.1
	+	+	Ethyl methyl ketone D	C_4H_8O	72.1
	-	+	1-penten-3-one M[2]	C_5H_8O	84.1
	-	+	Hydroxyacetone	$C_3H_6O_2$	74.1
	-	+	1-penten-3-one D	C_5H_8O	84.1
	+	-	3-methylbutanoic acid	$C_5H_{10}O_2$	102.1
	+	+	2-Methylpropionic acid	$C_4H_8O_2$	88.1
Acids	+	-	butanoic acid	$C_4H_8O_2$	88.1
	-	+	acetic acid	$C_2H_4O_2$	60.1
	-	+	propanoic acid	$C_3H_6O_2$	74.1
	+	+	ethyl propanoate	$C_5H_{10}O_2$	102.1
	+	+	Acetic acid, methyl ester	$C_3H_6O_2$	74.1
	+	-	Methyl 2-methylbutanoate	$C_6H_{12}O_2$	116.2
	+	+	Propyl acetate	$C_5H_{10}O_2$	102.1
Esters	+	-	3-Methylbutyl acetate	$C_7H_{14}O_2$	130.2
	+	+	Ethyl Acetate M[2]	$C_4H_8O_2$	88.1
	+	+	Ethyl Acetate D	$C_4H_8O_2$	88.1
	+	+	ethyl 2-methylpropanoate	$C_6H_{12}O_2$	116.2
	+	+	ethyl butanoate	$C_6H_{12}O_2$	116.2
	+	-	Tetrahydrofuran	C_4H_8O	72.1
	+	-	2-Ethylfuran	C_6H_8O	96.1
Others	+	+	3-Butenenitrile	C_4H_5N	67.1
	-	+	Benzeneamine	C_6H_7N	93.1
	-	+	2-Ethylpyrazine	$C_6H_8N_2$	108.1

+	-	Styrene	C_8H_8	104.2
-	+	2-Propenal	C_3H_4O	56.1

“M” for High load group; “L” for Low load group; M and D in the compound names respectively represent the two types of dimers of this substance; M^2 in the compound name indicates that the dimer M of this compound appears in the high load group.

Table S2. Signal intensities of volatile compounds detected by HS-GC-IMS in the high inoculation group.

No.	Compound	RI	RT	Dt	CAS	Signal intensity						
						ck	0h	4h	8h	12h	24h	48h
1	Cyclohexanone	887.1	373.435	1.4596	108-94-1	314.5090 ±44.7160	310.3923 ±19.7662	378.7023 ±9.8891	602.9780 ±43.1098	540.698 ±19.7461	349.8747 ±20.0460	42.8007 ±8.8658
2	3-methylbutanoic acid	887.3	373.717	1.2143	503-74-2	247.8977 ±12.6939	265.3140 ±11.1312	312.1950 ±10.0000	184.4280 ±5.5039	162.12 ±13.5324	78.2550 ±3.7753	33.7110 ±4.8528
3	2-Heptanone M	887.8	374.325	1.264	110-43-0	466.6450 ±79.8994	315.6197 ±34.1059	766.7630 ±4.2199	553.9163 ±14.3155	1185.148 ±28.3408	1716.6050 ±7.4304	324.0303 ±15.7241
4	Benzaldehyde	954.1	475.359	1.1538	100-52-7	143.1577 ±4.3957	203.9330 ±8.9431	268.6843 ±8.3844	279.2063 ±2.0754	178.685 ±14.9606	84.8620 ±2.7234	148.1643 ±23.9053
5	2-Methylpropionic acid	797.3	279.419	1.3562	79-31-2	844.6790 ±153.6683	727.3107 ±87.2508	1102.8990 ±94.4407	794.5523 ±25.4769	706.389 ±74.0334	234.7567 ±31.0727	476.6707 ±18.5338
6	3-Methyl-3-buten-1-ol	724.8	228.207	1.2884	763-32-6	56.1933 ±3.9131	57.5967 ±2.9258	62.3147 ±2.7676	62.5350 ±10.9947	175.7497 ±17.9966	602.8983 ±10.1958	390.9833 ±14.7582
7	Ethyl Acetate	608.4	175.526	1.3385	141-78-6	909.7747 ±20.2904	929.2090 ±20.9897	3533.807 ±36.7678	2424.6383 ±86.1539	4391.6973 ±46.4557	3282.5063 ±95.4514	404.9140 ±143.5869
8	2-Pentanone	681.3	205.008	1.3745	107-87-9	31.5163 ±2.7827	38.6143 ±0.7640	63.1080 ±2.3337	65.7113 ±4.7720	54.2823 ±4.4956	40.2010 ±2.3565	14.2717 ±0.9313
9	ethyl propanoate D	709	219.214	1.455	105-37-3	37.5293 ±5.1341	34.5017 ±5.3757	92.4567 ±2.0100	920.5800 ±58.0023	2655.0643 ±64.3209	1817.8207 ±77.7880	420.1043 ±65.4609

10	Tetrahydrofuran	620.2	179.629	1.2293	109-99-9	230.9633<br>±4.8953	229.215<br>±19.5046	275.3627<br>±9.9942	145.5697<br>±1.8091	249.5947<br>±34.9377	690.8500<br>±13.2711	679.8103<br>±50.8448
11	pentan-2,3-dione	692.7	210.6	1.2221	600-14-6	112.5470<br>±1.7891	93.9970<br>±3.5509	196.3503<br>±5.6836	298.3837<br>±32.6372	664.6943<br>±6.1572	455.1833<br>±1.6460	75.8873<br>±3.7442
12	3-methylbutanol M	733.1	233.17	1.2444	123-51-3	40.3413<br>±3.4968	70.8723<br>±3.9772	46.0847<br>±1.7105	209.5643<br>±18.4586	46.0843<br>±6.6722	143.8630<br>±10.5586	40.2097<br>±4.5835
13	3-methylbutanol D	732.9	233.045	1.4928	123-51-3	24.0093<br>±3.5785	32.1567<br>±4.1175	23.5197<br>±2.2169	308.9423<br>±46.5427	103.763<br>±4.0036	22.9867<br>±1.1637	45.8127<br>±4.7964
14	ethyl propanoate M	708.9	219.161	1.1523	105-37-3	53.9033<br>±8.7360	39.5903<br>±2.8970	219.2203<br>±10.2988	212.7520<br>±13.9692	224.2347<br>±13.9556	480.3717<br>±33.1244	178.4560<br>±15.6862
15	Acetoin	715.7	222.931	1.3337	513-86-0	27.1353<br>±6.2915	24.7500<br>±3.5251	33.8240<br>±4.0888	66.2633<br>±11.9371	40.191<br>±1.2025	24.0363<br>±3.4865	100.8567<br>±14.147
16	2-methylpropanol	623.9	180.941	1.1696	78-83-1	133.3920<br>±10.6978	112.2793<br>±4.6612	388.4847<br>±3.4511	256.5083<br>±1.9838	258.2973<br>±6.9578	560.6423<br>±15.5284	300.6200<br>±34.1575
17	Acetic acid, methyl ester	534.3	154.461	1.1893	79-20-9	48.1877<br>±3.6346	46.7820<br>±0.9667	112.9330<br>±1.2397	349.0860<br>±0.6010	251.703<br>±6.1205	46.1980<br>±1.6256	20.7377<br>±5.2781
18	1-Octen-3-ol	975	514.31	1.1608	339-18-6	63.9427<br>±2.5445	77.4577<br>±7.9390	167.0163<br>±6.9619	146.7370<br>±2.9001	202.6997<br>±25.0774	438.1660<br>±38.6611	1260.2220<br>±22.1834
19	1-Hexanol	871.7	354.299	1.6423	111-27-3	16.0933<br>±1.7603	20.6543<br>±3.9628	15.1643<br>±1.5748	72.7617<br>±5.2497	40.529<br>±6.0357	14.0443<br>±1.6214	15.3447<br>±2.415
20	2-Heptanone D	887.4	373.879	1.6284	110-43-0	22.9730<br>±2.5769	23.7403<br>±6.7582	23.4120<br>±1.8642	34.7423<br>±5.5078	108.6033<br>±3.1046	268.9860<br>±12.6699	21.2157<br>±1.0965
21	Methyl 2-methylbutanoate	779.4	265.076	1.1959	868-57-5	47.7507<br>±3.4840	48.1053<br>±5.7066	79.5963<br>±4.6452	140.6717<br>±14.5337	257.6597<br>±3.4053	281.0970<br>±4.4943	159.2790<br>±25.3982
22	3-Butenenitrile	659.8	195.196	1.2532	109-75-1	474.275<br>±34.9034	547.1480<br>±68.1583	288.1713<br>±12.3687	814.6990<br>±11.7019	862.9287<br>±16.1693	1491.8797<br>±84.5225	567.4503<br>±45.4542
23	Propyl acetate	702.9	215.909	1.1603	109-60-4	36.8067<br>±5.3047	23.1720<br>±2.5905	175.1433<br>±4.7157	91.4730<br>±9.9595	371.7063<br>±17.1787	878.3310<br>±49.8218	354.0890<br>±58.8561

24	ethyl 2-methylpropanoate D	759	250.157	1.5652	97-62-1	9.3080<br>±3.1749	8.3043<br>±1.9985	7.4167<br>±0.3079	8.7573<br>±1.4065	33.6943<br>±24.8187	8.1653<br>±0.5592	8.3073<br>±1.6544
25	3-(methylthio) propanal	901.7	392.943	1.4051	3268-49-3	27.4440<br>±6.0644	36.6560<br>±6.8367	34.7223<br>±9.9522	34.9307<br>±1.4082	73.5897<br>±23.937	63.0883<br>±11.0029	75.0310<br>±3.5173
26	Benzeneamine	974.7	513.772	1.4293	62-53-3	45.6233<br>±1.3912	45.5597<br>±4.7413	46.6417<br>±2.4809	49.6547<br>±6.6042	66.684<br>±3.1986	105.9413<br>±17.0177	932.7397<br>±45.1515
27	phenylacetaldehyde	1042	666.986	1.2521	122-78-1	72.3637<br>±8.9072	85.1400<br>±14.6785	74.4367<br>±2.6967	88.5437<br>±7.8457	126.338<br>±15.9672	219.1837<br>±8.4832	259.0677<br>±6.8858
28	ethyl 2-methylpropanoate M	753.5	246.39	1.1873	97-62-1	176.6353<br>±9.3262	200.7690<br>±7.7259	291.6123<br>±10.7912	451.5863<br>±9.6657	377.4213<br>±17.1283	441.2910<br>±12.0757	438.8603<br>±125.08
29	Hexyl acetate	1013.6	597.329	1.8926	142-92-7	438.5080<br>±10.2821	505.8317<br>±42.5996	526.5280<br>±26.7593	580.0563<br>±42.1493	631.7967<br>±333.3567	1629.7480<br>±370.7151	2571.3100<br>±1768.7938
30	3-Methylbutyl acetate	881.4	366.171	1.2964	123-92-2	47.7863<br>±5.3677	45.2457<br>±2.0866	53.0440<br>±4.4294	50.6167<br>±0.2389	81.899<br>±5.3025	138.2400<br>±5.4395	780.6300<br>±30.7885
31	Styrene	891	378.514	1.4106	100-42-5	32.8010<br>±3.9616	31.3020<br>±2.2884	35.1797<br>±0.2150	45.8893<br>±3.5998	52.2603<br>±2.7586	51.1910<br>±6.0714	184.8450<br>±22.7181
32	2-Ethylfuran	707.8	218.547	1.3158	3208-16-0	37.6740<br>±2.3572	29.6210<br>±3.0614	36.6357<br>±3.1442	115.0843<br>±7.7564	164.977<br>±7.3826	220.5827<br>±1.6057	1137.6240<br>±139.1571
33	butanoic acid	800.3	281.984	1.3765	107-92-6	192.9343<br>±46.6452	174.2230<br>±37.3580	568.3227<br>±53.3358	198.8507<br>±3.3475	257.2893<br>±35.2459	43.9477<br>±3.4662	974.5690<br>±89.0113
34	Ethyl methyl ketone	607.6	175.262	1.2579	78-93-3	102.1527<br>±6.5306	128.7163<br>±13.3812	167.4083<br>±6.1926	393.7317<br>±20.8394	218.159<br>±17.3849	485.6380<br>±14.0788	1212.2643<br>±201.2773

M and D respectively represent the two types of dimers of this substance.

Table S3. Signal intensities of volatile compounds detected by HS-GC-IMS in the low inoculation group..

No.	Compound	RI	RT	Dt	CAS	Signal intensity						
						CK	0h	4h	8h	12h	24h	48h
1	2-Propenal	412.5	132.671	1.0468	107-02-8	4611.6223 ±71.6547	4325.5363 ±30.7141	4884.5073 ±15.9680	4414.7443 ±137.6541	1898.2607 ±607.6934	367.4417 ±29.2834	216.1197 ±19.9409
2	Ethyl methyl ketone M	590.5	169.72	1.0602	78-93-3	331.9623 ±3.9636	326.3987 ±31.7128	405.2183 ±21.9153	304.5910 ±10.3538	82.1150 ±14.4172	22.0477 ±1.0932	13.5147 ±1.5941
3	Ethyl Acetate M	609.6	175.909	1.0972	141-78-6	629.1627 ±2.6240	574.3550 ±11.946	671.6867 ±62.2511	543.8243 ±10.7543	259.6053 ±31.0901	150.1703 ±13.0989	78.2940 ±6.4702
4	2-3-Butanedione	588.6	169.118	1.1687	431-03-8	201.3527 ±13.6855	206.6023 ±3.8771	251.8940 ±36.675	296.9427 ±16.6425	341.1160 ±23.2704	114.9207 ±8.8822	74.2480 ±2.5435
5	2-Methylbutanal D	660.1	195.349	1.3996	96-17-3	508.2160 ±119.7297	481.1597 ±122.4143	790.9353 ±84.6283	1056.9217 ±175.7797	510.0740 ±93.4563	151.6333 ±7.7503	15.8580 ±0.9359
6	3-Methylbutanal M	650.2	191.137	1.1725	590-86-3	384.5277 ±7.8883	398.4777 ±23.8132	404.5137 ±57.6656	207.6957 ±28.2624	134.8363 ±3.4326	58.6603 ±0.9991	25.6723 ±1.5703
7	3-Butenenitrile	659.9	195.23	1.2506	109-75-1	1283.7747 ±125.9392	1287.9140 ±156.6991	1403.1983 ±138.8621	2007.2330 ±42.5256	2897.5943 ±58.5922	2428.6820 ±97.3117	1427.2517 ±18.0152
8	Butanoic acid methyl ester	718.2	224.357	1.1597	623-42-7	153.6393 ±31.8507	185.0753 ±35.4024	140.8630 ±15.6775	198.9033 ±15.9448	417.3070 ±75.3729	449.4373 ±26.7662	162.4223 ±9.3873
9	Ethyl 2-methylpropanoate	739	236.883	1.2016	97-62-1	61.3560 ±1.4785	41.3137 ±2.3294	28.5140 ±1.6175	47.7190 ±4.6530	76.2937 ±14.7982	36.2507 ±2.5201	15.0823 ±1.4663
10	1-Pentanol M	766.9	255.775	1.2527	71-41-0	481.2537 ±1.7681	226.4503 ±27.3511	516.1897 ±49.3976	223.3413 ±11.5083	193.2840 ±41.7564	92.8653 ±2.1378	31.4467 ±5.1631
11	Hexanal	797.5	279.608	1.2551	66-24-1	968.6983 ±35.7765	732.8823 ±230.024	1110.5563 ±39.7487	497.7907 ±177.9647	306.0163 ±53.3683	149.8927 ±11.0870	79.0110 ±2.0688
12	3-Methylthio propanal	902.3	393.824	1.0908	3268-49-3	118.4790 ±20.6855	98.8250 ±43.6363	235.2537 ±26.2648	243.3230 ±18.9153	193.6140 ±36.7771	93.0350 ±13.0763	31.0637 ±8.9408

13	2-Ethylpyrazine	887.7	374.205	1.1572	13925-00-3	356.8733<br>±4.2428	419.0243<br>±10.3485	1165.0070<br>±84.8196	1155.1187<br>±42.7615	627.1817<br>±86.3321	553.4107<br>±31.8320	345.0427<br>±8.1622
14	Ethyl pentanoate	887.6	374.037	1.2692	539-82-2	366.2640<br>±9.8328	331.4580<br>±3.5671	457.0077<br>±20.6239	790.7883<br>±62.7999	1196.5080<br>±92.6197	1286.5570<br>±130.6111	2053.9783<br>±38.6351
15	Benzaldehyde	954.2	475.42	1.1532	100-52-7	230.5770<br>±21.1791	328.5617<br>±47.9766	393.8390<br>±32.7856	349.9897<br>±25.2377	265.5300<br>±26.3150	135.3027<br>±5.9969	142.4203<br>±6.0027
16	2-Propanol	513.5	149.77	1.0873	67-63-0	1480.6877<br>±63.1524	1591.6703<br>±34.3535	1121.2683<br>±12.5007	1649.2590<br>±136.9787	4037.4693<br>±746.1977	3602.6660<br>±223.5030	1116.1920<br>±47.8270
17	1-Butanol	665	197.486	1.1797	71-36-3	171.5370<br>±27.1028	153.3493<br>±33.7962	209.3113<br>±9.6238	122.6420<br>±10.0777	51.1300<br>±3.2851	72.2307<br>±4.3928	22.5103<br>±2.8960
18	3-Methylbutanol M	732.8	233.006	1.2426	123-51-3	80.3790<br>±5.1282	62.5410<br>±8.6151	87.4733<br>±12.38	91.2917<br>±23.1773	60.5263<br>±7.0481	50.3413<br>±8.3331	21.7677<br>±3.9994
19	Cyclohexanone	887.5	373.917	1.4607	108-94-1	69.8743<br>±2.5645	83.3050<br>±3.5448	547.7737<br>±118.3956	652.0753<br>±20.8855	335.7100<br>±70.2870	336.4613<br>±70.0852	267.0603<br>±5.7466
20	1-Pentanol D	766.4	255.362	1.5081	71-41-0	59.8147<br>±2.1474	12.7077<br>±3.3377	59.4777<br>±14.238	14.5653<br>±1.1187	20.0500<br>±6.7573	6.2407<br>±0.4716	4.6600<br>±1.0143
21	1-Octen-3-ol	975.2	514.759	1.1605	3391-86-4	172.8117<br>±20.6839	160.4537<br>±14.2177	398.4597<br>±31.4688	675.3523<br>±18.7950	679.6597<br>±41.0168	785.4847<br>±87.2318	1972.2027<br>±55.4782
22	Trans-2-pentenal	738.9	236.813	1.3522	1576-87-0	18.0237<br>±3.1912	9.7093<br>±0.3857	35.8700<br>±4.9548	30.4487<br>±4.1353	16.4647<br>±4.0400	8.4257<br>±0.2026	5.3023<br>±0.8073
23	2-Heptanone	887.1	373.409	1.6271	110-43-0	26.7363<br>±1.9508	23.8607<br>±1.1783	38.4077<br>±2.2129	77.6157<br>±3.5727	126.0200<br>±10.8682	147.6163<br>±30.4637	500.8237<br>±2.3923
24	3-Methylbutanol D	733.1	233.177	1.4949	123-51-3	149.7323<br>±10.8548	135.6517<br>±20.1316	163.7643<br>±34.4205	210.3827<br>±49.7670	1512.3337<br>±228.8086	1774.0783<br>±36.7410	235.2827<br>±20.5091
25	3-Methyl-3-buten-1-ol	733.1	233.209	1.278	763-32-6	232.2010<br>±21.2684	237.2963<br>±50.6155	231.1930<br>±22.6900	223.9543<br>±28.0868	1064.2543<br>±194.7732	1658.1850<br>±56.8033	1683.5860<br>±4.7803
26	1-Propanol	551.8	158.776	1.2551	71-23-8	48.2840<br>±10.6296	42.8263<br>±5.1588	52.2073<br>±3.7056	56.4520<br>±6.6401	1199.5823<br>±322.4857	1002.3157<br>±58.3706	111.2990<br>±5.3129

27	Acetic acid methyl ester	537.8	155.304	1.1872	79-20-9	49.3157 ±7.5222	41.0610 ±5.1433	46.3803 ±5.0456	89.7033 ±7.6894	171.2263 ±31.4604	115.3170 ±3.2984	16.8267 ±1.2437
28	Ethyl propanoate	704.7	216.883	1.455	105-37-3	15.3280 ±1.6862	16.9213 ±1.9347	16.0983 ±1.6641	16.7070 ±0.9714	23.0770 ±3.4986	1126.4810 ±62.1180	23.4440 ±1.3323
29	Ethyl butanoate	781.8	266.922	1.2068	105-54-4	35.5900 ±3.0097	32.0103 ±3.0347	27.0993 ±2.7045	73.0210 ±6.4246	88.2930 ±7.7375	160.1690 ±21.631	158.1220 ±8.5675
30	1-penten-3-one M	688.7	208.61	1.0785	1629-58-9	14.6700 ±1.4903	18.2490 ±1.5533	14.3290 ±0.7875	16.3760 ±0.7098	15.3510 ±3.1368	45.2103 ±5.2258	26.1147 ±2.1275
31	Hydroxyacetone	627.3	182.184	1.0435	116-09-6	991.2147 ±23.9461	959.2800 ±52.8669	1068.8833 ±85.4168	1113.2750 ±62.6510	665.6483 ±113.9654	498.1603 ±26.6480	101.4503 ±3.2805
32	Benzeneamine	974.1	512.625	1.429	62-53-3	19.1047 ±1.7858	17.6060 ±0.9414	21.0227 ±5.5420	33.1160 ±1.4471	32.9007 ±4.5245	33.1887 ±9.5356	179.4317 ±12.2215
33	Heptanal	885.7	371.623	1.3259	111-71-7	55.8313 ±0.2679	28.1537 ±4.8271	48.9453 ±3.9205	44.6913 ±4.696	62.4553 ±2.9979	53.3940 ±4.8278	824.8853 ±8.8133
34	E-2-Hexenal	834	313.049	1.1886	6728-26-3	24.8897 ±1.5943	21.9107 ±1.1263	24.7113 ±1.2345	18.5217 ±1.4347	28.7487 ±3.8754	28.4213 ±1.8536	95.5763 ±2.2695
35	2-Methylpropionic acid	797.9	279.891	1.3772	79-31-2	31.7687 ±3.3441	20.2597 ±9.9379	25.2130 ±4.3194	13.6383 ±3.7633	12.8377 ±2.3307	7.74370 ±0.7114	67.9080 ±1.7782
36	Pentan-2-3-dione	676.1	202.534	1.2242	600-14-6	36.7790 ±2.0316	35.3550 ±2.4428	19.8253 ±2.4125	27.6940 ±3.1567	71.1433 ±4.9700	249.4083 ±16.2032	151.5687 ±12.6790
37	1-penten-3-one D	688.4	208.466	1.3108	1629-58-9	8.9277 ±0.6574	5.6307 ±1.0383	8.7073 ±0.9109	5.6653 ±1.5017	12.2800 ±3.3370	24.3047 ±4.4594	138.3377 ±17.9104
38	2-Hexanol	776.9	263.153	1.2817	626-93-7	12.7827 ±0.8878	7.3797 ±1.6738	9.5827 ±1.2675	9.5517 ±0.8470	26.5703 ±1.1585	23.5267 ±3.0930	89.4720 ±1.7144
39	Dimethyl ketone	492.4	145.501	1.1178	67-64-1	6662.4570 ±303.5526	9446.1217 ±198.8762	9029.9290 ±522.238	7955.7057 ±579.5322	4374.1783 ±1215.6313	3154.3453 ±95.8408	791.0320 ±8.8871
40	Ethyl methyl ketone D	589.3	169.336	1.2478	78-93-3	558.2137 ±11.8910	671.4220 ±17.3094	1001.5257 ±52.7438	844.8480 ±59.8986	474.5470 ±142.5509	63.9847 ±10.1894	338.0943 ±12.8102

41	Ethyl Acetate D	609.2	175.792	1.3381	141-78-6	1397.6410 ±37.2963	904.7640 ±20.1754	2683.0717 ±41.1027	2864.0593 ±44.8678	2349.3443 ±383.3214	1148.5680 ±7.6038	47.1397 ±4.9142
42	Acetic acid	589	169.248	1.1526	64-19-7	511.8660 ±0.8856	448.2257 ±15.3966	322.5187 ±7.1527	634.7643 ±66.3872	2573.5940 ±397.3932	1893.2330 ±124.0187	2319.9917 ±115.4139
43	2-Methylbutanal M	660.8	195.637	1.1595	96-17-3	241.7077 ±33.7497	258.1317 ±41.0617	231.0230 ±20.2286	153.3880 ±10.5490	108.5713 ±0.5520	120.8407 ±34.1695	165.6853 ±12.8397
44	3-Methylbutanal D	649.9	191.041	1.4082	590-86-3	554.8707 ±129.3029	485.3167 ±169.2504	1258.7260 ±95.3785	1541.3567 ±172.9235	728.4330 ±101.2454	335.6997 ±29.5957	24.7023 ±0.5292
45	2-Pentanone	682.4	205.509	1.3728	107-87-9	11.7237 ±0.4153	17.9060 ±1.4972	25.8240 ±3.0204	12.3530 ±2.2576	17.2610 ±4.1952	9.6940 ±1.0221	4.42700 ±0.3618
46	Pentanal	692.3	210.431	1.1838	110-62-3	114.8503 ±12.5670	78.5733 ±23.8983	123.2607 ±9.48	40.0103 ±11.3438	26.4487 ±3.1894	42.7700 ±1.7530	74.8853 ±3.1666
47	Propanoic acid	692.1	210.303	1.2634	79-09-4	64.4497 ±9.4115	37.0653 ±25.1128	39.4223 ±3.1877	31.0827 ±9.9399	133.1713 ±27.7847	37.5083 ±3.4761	67.9157 ±3.0749
48	Acetoin	710.5	220.02	1.3326	513-86-0	19.8230 ±2.3552	22.9207 ±8.0391	86.6620 ±15.0827	137.1557 ±23.7720	181.1490 ±57.5342	90.4610 ±9.2099	67.3743 ±4.0269
49	Propyl acetate	698.2	213.427	1.1587	109-60-4	125.1937 ±8.1840	85.2577 ±8.2609	193.1777 ±14.0026	450.4543 ±28.1991	1231.4143 ±79.4392	1511.2290 ±90.8536	1054.894 ±26.7168

M and D respectively represent the two types of dimers of this substance.

Table S4. List of VOCs identified using HS-SPME-GC-MS in *E. coli*-inoculated chicken meat stored for 0-48 h.

Class	Compound	CAS	RT.(min)	Formula	RI	LRI
Alcohols	3-Methylbutanol	123-51-3	3.871	$C_5H_{12}O$	736	734
	2-Methylbutanol	137-32-6	3.96	$C_5H_{12}O$	739	738
	1-Hexanol	111-27-3	8.114	$C_6H_{14}O$	868	872
	2-Heptanol	543-49-7	9.42	$C_7H_{16}O$	900	901
	1-Octen-3-ol	3391-86-4	13.041	$C_8H_{16}O$	980	981
	2-Octanol*	123-96-6	14.091	$C_8H_{18}O$	998	1000
	2-Ethylhexanol	104-76-7	15.272	$C_8H_{18}O$	1030	1032
	2,4-Dimethylcyclohexanol	69542-91-2	15.397	$C_8H_{16}O$	1032	1035
	(2E)-2-Octen-1-ol	18409-17-1	16.86	$C_8H_{16}O$	1067	1070
	Phenylethyl alcohol	60-12-8	18.422	$C_8H_{10}O$	1116	1107
	1-Methylverbenol	——	22.864	$C_{11}H_{18}O$	——	1242
Aldehydes	Hexanal	66-25-1	5.58	$C_6H_{12}O$	800	801
	Heptanal	111-71-7	8.114	$C_7H_{14}O$	901	901
	Benzaldehyde	100-52-7	11.978	C_7H_6O	962	960
	Phenylacetaldehyde	122-78-1	15.789	C_8H_8O	1045	1045
	Nonanal	124-19-6	18.199	$C_9H_{18}O$	1104	1100
	Decanal	112-31-2	21.623	$C_{10}H_{20}O$	1206	1200
	Hexadecanal	629-80-1	36.622	$C_{16}H_{32}O$	1817	1814
Ketones	2-Heptanone	110-43-0	8.969	$C_7H_{14}O$	891	891
	2-Octanone	111-13-7	13.576	$C_8H_{16}O$	990	991
	Bicyclo[3.2.0]hept-3-en-2-one	19093-11-9	14.89	C_7H_8O	——	1022
	2-Nonanone	821-55-6	17.74	$C_9H_{18}O$	1092	1090
	Isophorone	78-59-1	18.697	$C_9H_{14}O$	1124	1116
	3,8-Nonadien-2-one	872858-42-9	23.689	$C_{11}H_{18}O$	1276	1269
	(Z)-Undec-6-en-2-one	107853-70-3	23.784	$C_{11}H_{20}O$	1274	1272
Acids	3-Methylbutanoic acid	503-74-2	7.075	$C_5H_{10}O_2$	863	846
	2-Methylbutanoic acid	116-53-0	7.55	$C_5H_{10}O_2$	861	858
	n-Hexadecanoic acid	57-10-3	38.688	$C_{16}H_{32}O_2$	1968	1958
	(2E)-2-(Hydroxyimino)-3-phenylpropanoic acid	3682-17-5	19.386	$C_9H_9NO_3$	——	1137
Esters	Ethyl tiglate	5837-78-5	11.195	$C_7H_{12}O_2$	939	944
	2,2,2-Trifluoroethyl (9E)-9-octadecenoate	——	33.322	$C_{20}H_{39}F_3O_2$	——	1647
Hydrocarbon	2,4-Dimethylheptane	2213-23-2	6.269	C_9H_{20}	821	823
	D-Limonene	5898-27-5	15.13	$C_{10}H_{16}$	——	1028
	4-Methyldecane	2847-72-5	16.531	$C_{11}H_{24}$	1060	1063
	Undecane	1120-21-4	18.035	$C_{11}H_{24}$	1100	1096
	Dodecane	112-40-3	21.433	$C_{12}H_{26}$	1200	1194
	Tetradecane	629-59-4	27.155	$C_{14}H_{30}$	1400	1391
	Pentadecane	629-62-9	19.684	$C_{15}H_{32}$	1500	1489
	Hexadecane	544-76-3	32.064	$C_{16}H_{34}$	1600	1590

	Ethylbenzene	100-41-4	7.687	C_8H_{10}	855	862
	Styrene	100-42-5	8.862	C_8H_8	893	889
	2-Ethyl-1,4-dimethylbenzene	1758-88-9	17.245	$C_{10}H_{14}$	1074	1079
	1-Ethyl-2,4-dimethylbenzene	874-41-9	17.148	$C_{10}H_{14}$	1075	1077
	1,2,4,5-Tetramethylbenzene	95-93-2	18.513	$C_{10}H_{14}$	1116	1107
	1-Vinyl-1H-indene	2471-83-2	24.674	$C_{11}H_{10}$	1315	1315
Nitrogen and sulfur compound	2-Methylbutylamine	96-15-1	3.61	$C_5H_{13}N$	701	722
	Methyl N-hydroxybenzenecarboximidate	——	9.8	$C_8H_9NO_2$	——	911
	Indole	120-72-9	24.211	C_8H_7N	1295	1286
	Cyclic octaatomic sulfur	10544-50-0	39.748	S_8	2049	2045
Others	2,4-Di-tert-butylphenol	96-76-4	30.004	$C_{16}H_{34}$	1570	1561
	2,3-Epoxy-4,4-dimethylpentane	53897-30-6	3.894	$C_7H_{14}O$	——	735

*:2-Octanol used as internal standard; LRI queried with C_7-C_{40} n-alkane stander.

Table S5. Changes in volatile compounds during chicken meat storage (mean ± SD) and correlation analysis with storage time in the high inoculation level group of *E. coli.*

Metabolite	ck	0h	4h	8h	12h	24h	48h	Coefficient of correlation (r)
2-Methylbutylamine	ND	ND	ND	ND	ND	ND	3.3772±0.0136	0.879
2-Methyl-3-(2-methyl-2-propanyl)oxirane	ND	0.1518±0.0220	0.1283±0.0222	0.1150±0.0283	ND	ND	ND	-0.552
3-Methylbutanol	ND	ND	ND	ND	1.1555±0.0351	1.2504±0.0163	4.6948±0.1766	0.967
2-Methylbutanol	ND	ND	ND	ND	0.3629±0.0431	0.3028±0.0020	0.4660±0.0637	0.860
Hexanal	2.2144±0.0085	1.3173±0.0344	1.2767±0.0136	1.4228±0.0079	2.3501±0.0228	0.5754±0.0057	ND	-0.772
2,4-Dimethylheptane	ND	ND	0.6729±0.0035	0.1614±0.0023	0.3539±0.0372	0.2400±0.0223	0.3606±0.0190	0.268
3-Methylbutanoic acid	ND	ND	ND	ND	ND	ND	6.2723±0.0476	0.879
2-Methylbutanoic acid	ND	ND	ND	ND	ND	ND	2.0597±0.0165	0.879
Ethylbenzene	ND	0.0900±0.0088	ND	ND	ND	ND	ND	-0.357
1-Hexanol	ND	0.3195±0.0007	0.2954±0.0016	ND	1.1676±0.0421	0.7684±0.0341	ND	-0.037
Styrene	ND	0.0959±0.0007	ND	ND	ND	ND	ND	-0.357
2-Heptanone	ND	ND	ND	ND	0.3750±0.0104	3.3294±0.0194	3.1956±0.0184	0.886
2-Heptanol	ND	ND	ND	ND	ND	ND	0.4331±0.0490	0.879
Heptanal	0.1859±0.0016	ND	0.0884±0.0010	0.0899±0.0029	0.3556±0.0041	0.4292±0.0309	ND	-0.005
Methyl N-hydroxybenzenecarboximidate	15.7211±0.0322	14.8381±0.1124	16.9132±0.006	14.7888±0.1506	19.5807±0.0063	25.1397±0.1298	34.8180±0.1508	0.983
Ethyl tiglate	ND	ND	ND	ND	ND	4.7852±0.0549	4.7722±0.0793	0.882
1-Octen-3-ol	0.5665±0.0023	0.9377±0.0199	0.9568±0.0245	1.0847±0.0088	2.1141±0.0193	1.9662±0.0193	0.2445±0.0174	-0.165
2-Octanone	3.5378±0.0150	3.2064±0.1054	2.3113±0.1583	2.2263±0.0428	6.3628±0.2342	11.7842±0.1646	18.2136±0.0817	0.970
Bicyclo[3.2.0]hept-3-en-2-one	ND	ND	ND	ND	ND	ND	1.9411±0.0110	0.879
D-Limonene	0.5644±0.0028	ND	ND	ND	ND	ND	ND	-0.331

2-Ethylhexanol	0.6417±0.0049	2.4863±0.3255	0.9385±0.0029	0.9317±0.0278	1.9381±0.0832	1.558±0.0297	0.7315±0.0085	-0.283
Phenylacetaldehyde	ND	ND	ND	0.0836±0.0057	0.3825±0.0148	0.4810±0.0047	0.5453±0.0328	0.881
4-Methyldecane	ND	ND	0.2334±0.0440	0.1521±0.0177	ND	ND	ND	-0.320
(2E)-2-Octen-1-ol	ND	ND	ND	ND	ND	0.3237±0.1115	ND	0.261
2-Ethyl-1,4-dimethylbenzene	ND	ND	0.5520±0.0086	0.5335±0.0880	0.7135±0.0033	0.5397±0.0025	0.5399±0.0295	0.474
1-Ethyl-2,4-dimethylbenzene	ND	ND	ND	0.4070±0.0020	0.7772±0.0218	0.4856±0.0045	0.4532±0.0275	0.524
2-Nonanone	ND	ND	ND	ND	ND	8.0385±0.0096	13.7108±0.2038	0.963
Undecane	ND	0.3414±0.0065	0.4870±0.0265	ND	0.6414±0.0211	ND	ND	-0.378
Nonanal	1.1316±0.0161	1.3911±0.0028	0.5997±0.0069	ND	ND	ND	ND	-0.641
Phenylethyl alcohol	ND	ND	ND	ND	ND	ND	6.7821±0.1591	0.879
1,2,4,5-Tetramethylbenzene	0.1951±0.0032	0.4084±0.0449	0.8607±0.0278	0.8675±0.0676	1.1702±0.0103	1.0361±0.0372	1.3186±0.1042	0.761
Isophorone	ND	ND	ND	ND	ND	6.4364±0.0191	8.6328±0.0448	0.939
(2E)-2-(Hydroxyimino)-3-phenylpropanoic acid	ND	ND	ND	0.0297±0.0028	0.0382±0.0035	ND	ND	-0.142
Dodecane	0.7646±0.0036	0.7152±0.0228	1.6451±0.0022	1.1655±0.1333	1.5132±0.0120	1.3663±0.0157	1.5307±0.0297	0.532
Decanal	0.3316±0.0016	0.2017±0.0419	ND	ND	ND	ND	ND	-0.507
1-Methylverbenol	ND	ND	ND	ND	ND	15.7495±0.2420	3.4752±0.1188	0.461
(Z)-Undec-6-en-2-one	ND	ND	ND	ND	ND	0.4657±0.0303	ND	0.261
Indole	ND	ND	ND	ND	ND	5.1194±0.1800	195.4522±1.8552	0.889
1-Vinyl-1H-indene	ND	ND	ND	ND	0.3068±0.0018	0.3119±0.0095	0.3661±0.0472	0.832
Tetradecane	0.7163±0.0097	0.4842±0.0336	0.8892±0.0740	0.6336±0.0055	0.8248±0.0276	0.8166±0.0026	1.0343±0.0178	0.743
Pentadecane	2.6634±0.0291	2.1675±0.1151	2.3621±0.0083	2.9116±0.0203	5.3006±0.0333	3.7457±0.1696	3.3091±0.0093	0.332
2,4-Di-tert-butylphenol	0.2344±0.0007	ND	ND	ND	ND	ND	ND	-0.331
Hexadecane	3.2743±0.0078	1.8162±0.0128	1.7023±0.0205	0.6288±0.0213	2.6464±0.0953	3.0031±0.0471	2.1679±0.0525	0.128
2,2,2-Trifluoroethyl (9E)-9-octadecenoate	ND	ND	ND	ND	1.1925±0.1642	ND	ND	-0.48
Hexadecanal	ND	ND	ND	ND	ND	1.5159±0.0227	ND	0.261

n-Hexadecanoic acid	0.0678±0.0013	ND	ND	0.0281±0.0008	ND	0.1817±0.0014	0.2375±0.0097	0.891
Cyclic octaatomic sulfur	ND	ND	ND	ND	ND	ND	0.6430±0.0340	0.879

VOCs that were not detected in individual samples were recorded as ND (not detected), indicating that the signal was below the analytical detection capability rather than representing absolute absence of the compound.

Table S6. Changes in volatile compounds during chicken meat storage (mean ± SD) and correlation analysis with storage time in the low inoculation level group of *E. coli*.

Metabolite	ck	0h	4h	8h	12h	24h	48h	Coefficient of correlation (r)
2-Methylbutylamine	ND	ND	ND	ND	ND	ND	2.7080±0.3533	0.879
3-Methylbutanol	ND	ND	ND	ND	0.7726±0.0122	3.8245±0.0019	2.4580±0.0044	0.768
2-Methylbutanol	ND	ND	ND	ND	0.1860±0.0032	1.2530±0.0033	0.6480±0.0240	0.694
Hexanal	2.2126±0.0081	0.8221±0.0024	0.6891±0.0075	1.1547±0.0037	1.9744±0.0084	0.9311±0.0088	ND	-0.608
2,4-Dimethylheptane	ND	0.1268±0.0087	0.4428±0.0061	0.2049±0.0040	0.2236±0.0044	0.2657±0.0038	0.2821±0.0019	0.325
3-Methylbutanoic acid	ND	ND	ND	ND	ND	0.5316±0.0002	1.4351±0.0033	0.969
2-Methylbutanoic acid	ND	ND	ND	ND	ND	0.5253±0.0043	1.0669±0.0157	0.970
Ethylbenzene	ND	0.0557±0.0025	ND	ND	ND	ND	ND	-0.357
1-Hexanol	ND	0.3589±0.0230	ND	0.2693±0.0154	0.6654±0.0053	0.8110±0.0010	ND	0.013
Styrene	ND	0.0607±0.001	ND	ND	ND	ND	ND	-0.357
2-Heptanone	ND	ND	ND	ND	ND	3.3125±0.5586	4.5736±0.0041	0.943
2-Heptanol	ND	ND	ND	ND	ND	ND	0.4339±0.0018	0.879
Heptanal	0.1592±0.0470	ND	ND	ND	0.9254±0.0041	0.5967±0.0103	ND	0.06
Methyl N-hydroxybenzenecarboximidate	17.6900±0.1657	16.5197±0.0111	14.8877±0.0406	14.4592±0.4352	18.4576±0.0139	28.7397±0.016	27.0971±0.0270	0.828
Ethyl tiglate	ND	ND	ND	ND	ND	3.1547±0.0883	4.4785±0.0039	0.946
Benzaldehyde	ND	ND	0.1536±0.0416	0.2044±0.0077	0.2942±0.0817	0.4873±0.3089	ND	0.089
1-Octen-3-ol	0.8142±0.0066	0.8268±0.0106	0.5046±0.0023	1.2102±0.0189	1.4888±0.0385	3.0931±0.0064	0.4118±0.0008	0.092
2-Octanone	3.1299±0.0143	3.0513±0.0223	3.6705±0.2185	3.5945±0.2651	25.6137±0.0280	16.7225±0.0085	13.9303±0.1260	0.498
Bicyclo[3.2.0]hept-3-en-2-one	ND	ND	ND	ND	ND	ND	2.1343±0.0202	0.879
D-Limonene	0.5905±0.0053	0.2670±0.0596	ND	ND	ND	ND	ND	-0.480

2-Ethylhexanol	0.7159±0.0013	1.0823±0.0223	1.6276±0.0195	0.8480±0.0288	1.5678±0.0015	0.8108±0.0766	0.7634±0.0022	-0.349
2,4-Dimethylcyclohexanol	ND	ND	ND	ND	ND	0.1746±0.0778	ND	0.261
Phenylacetaldehyde	ND	ND	ND	ND	ND	0.2981±0.0003	0.4838±0.0136	0.959
(2E)-2-Octen-1-ol	ND	ND	ND	ND	ND	0.3178±0.0378	ND	0.261
2-Ethyl-1,4-dimethylbenzene	ND	ND	0.6455±0.0121	0.4912±0.0035	0.5778±0.0020	0.5260±0.0018	0.7849±0.0052	0.685
1-Ethyl-2,4-dimethylbenzene	ND	ND	ND	ND	0.4745±0.0031	0.5030±0.0045	0.6751±0.0019	0.881
2-Nonanone	ND	ND	ND	ND	ND	11.4966±0.0076	11.5580±0.6671	0.884
Undecane	ND	ND	0.6421±0.0044	0.2549±0.0424	0.4765±0.0008	0.5958±0.0035	ND	-0.089
Nonanal	1.1279±0.0187	0.7600±0.0853	1.5786±0.0006	1.0440±0.0468	1.3475±0.0009	0.7955±0.0023	ND	-0.786
Phenylethyl alcohol	ND	ND	ND	ND	ND	ND	5.2792±0.0149	0.879
1,2,4,5-Tetramethylbenzene	0.1934±0.0060	0.3540±0.0130	0.8976±0.0150	0.7598±0.0075	0.8698±0.0148	1.1526±0.0769	1.8308±0.0022	0.938
Isophorone	ND	ND	ND	ND	ND	6.2641±0.0034	10.8462±0.0257	0.964
(2E)-2-(Hydroxyimino)-3-phenylpropanoic acid	ND	0.0495±0.0119	ND	0.0389±0.0067	0.0263±0.0034	ND	ND	-0.435
Dodecane	0.7646±0.0036	0.7214±0.0159	1.4303±0.0052	0.8934±0.0121	1.2562±0.0068	1.3883±0.0636	1.4707±0.0064	0.676
Decanal	0.3316±0.0016	0.3046±0.0019	ND	ND	ND	ND	ND	-0.531
1-Methylverbenol	ND	ND	ND	ND	ND	14.5064±0.0031	5.3754±0.0039	0.583
3,8-Nonadien-2-one	ND	ND	ND	ND	ND	0.7565±0.1411	ND	0.261
(Z)-Undec-6-en-2-one	ND	ND	ND	ND	ND	0.6262±0.0024	ND	0.261
Indole	ND	ND	ND	ND	ND	5.4903±0.0074	91.7801±0.4127	0.902
1-Vinyl-1H-indene	ND	ND	ND	ND	ND	0.3181±0.0070	0.4268±0.0437	0.939
Tetradecane	0.7297±0.0047	0.5486±0.0291	0.8190±0.0087	0.5654±0.0311	0.8659±0.0055	0.8638±0.0209	0.6268±0.0044	0.013
Pentadecane	2.6965±0.0021	1.9489±0.0363	2.5360±0.0833	2.8812±0.1116	3.0468±0.0117	4.4474±0.0730	2.1641±0.0058	0.117
2,4-Di-tert-butylphenol	0.2356±0.0016	ND	ND	ND	ND	ND	ND	-0.331
Hexadecane	3.2766±0.0117	1.1911±0.0068	2.4227±0.0027	3.4673±0.0051	3.1095±0.0177	2.5943±0.0921	1.6716±0.0336	-0.289
2,2,2-Trifluoroethyl (9E)-9-octadecenoate	ND	ND	ND	ND	0.0964±0.0363	ND	ND	-0.048

Hexadecanal	ND	ND	ND	ND	ND	2.5879±0.0032	ND	0.261
n-Hexadecanoic acid	0.0712±0.0076	0.0632±0.0046	0.0416±0.0055	ND	0.0374±0.0014	0.1761±0.0028	0.1993±0.0218	0.824
Cyclic octaatomic sulfur	ND	ND	ND	ND	ND	0.4270±0.0472	0.5995±0.0270	0.945

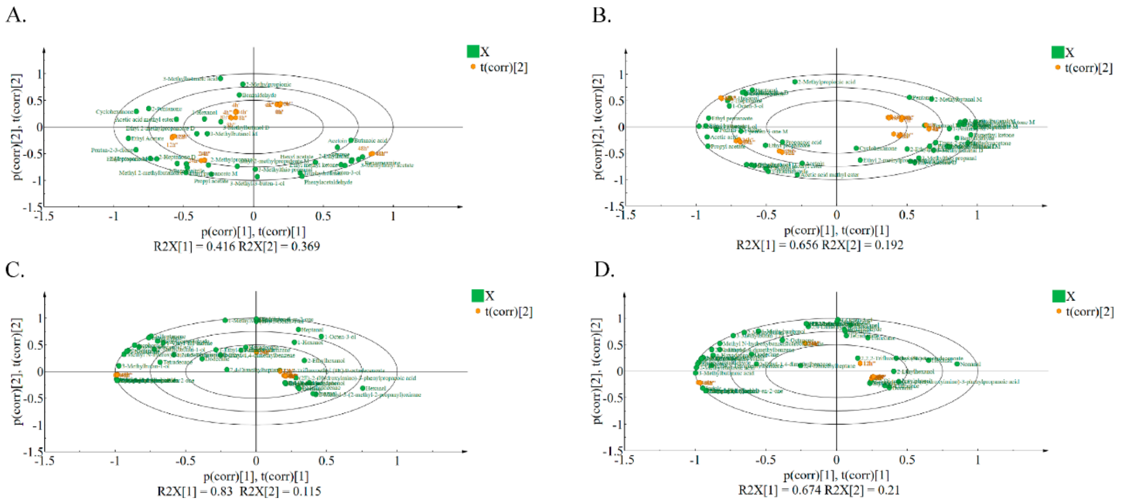

Figure S1. Orthogonal partial least squares-discriminant biplots. (A) OPLS-DA biplot of high inoculation group by HS-GC-IMS; (B) OPLS-DA biplot of low inoculation group by HS-GC-IMS; (C) OPLS-DA biplot of high inoculation group by HS-SPME-GC-MS; (D) OPLS-DA biplot of low inoculation group by HS-SPME-GC-MS.

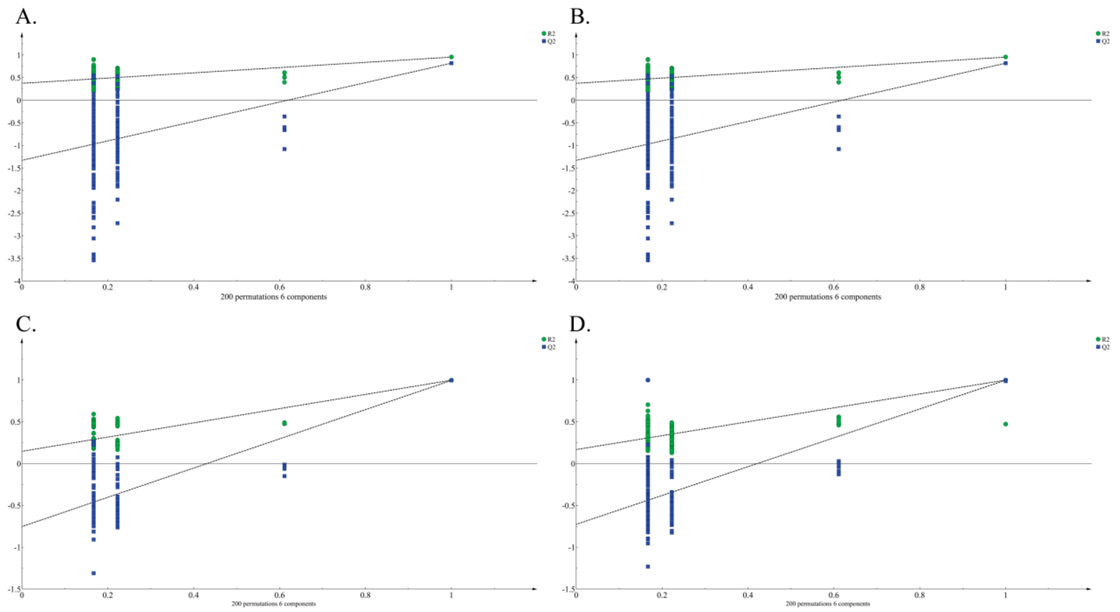

Figure S2. Permutation analysis. (A) Permutation analysis of high inoculation group by HS-GC-IMS; (B) Permutation analysis of low inoculation group by HS-GC-IMS; (C) Permutation analysis of high inoculation group by HS-SPME-GC-MS; (D) Permutation analysis of low inoculation group by HS-SPME-GC-MS.
